# Effectiveness and potential mechanism of Jiawei-Xiaoyao-San for hyperthyroidism: a systematic review

**DOI:** 10.3389/fendo.2023.1241962

**Published:** 2023-09-13

**Authors:** Wenxin Ma, Xiaowen Zhang, Ruotong Zhao, Yang Tang, Xiaoyun Zhu, Longkun Liu, Mingyuan Xu, Ge Wang, Peiyue Peng, Jianping Liu, Zhaolan Liu

**Affiliations:** ^1^ Centre for Evidence-Based Chinese Medicine, Beijing University of Chinese Medicine, Beijing, China; ^2^ Beijing University of Chinese Medicine, Beijing, China; ^3^ Guang’anmen Hospital, China Academy of Chinese Medical Sciences, Beijing, China; ^4^ Guang’anmen Hospital South Campus, China Academy of Chinese Medical Sciences, Beijing, China; ^5^ Department of Community Medicine, Faculty of Health Science, UiT The Arctic University of Norway, Tromsø, Norway

**Keywords:** hyperthyroidism, Jiawei-Xiaoyao-San, traditional Chinese medicine, systematic review, meta-analysis, randomized controlled trials, pharmacological studies

## Abstract

**Objectives:**

To evaluate the effectiveness and potential mechanism of traditional Chinese medicine Jiawei-Xiaoyao-San (JWXYS) as an adjunct or mono- therapy for antithyroid drugs (ATDs) in the treatment of hyperthyroidism.

**Methods:**

Eight databases and three trial registries were searched from inception until May 2023. Randomized controlled trials (RCTs) were included and meta-analysis was conducted using RevMan 5.4 and Stata 14.0. The Cochrane risk of bias (ROB) tool 1.0 and GRADE tool was used for quality appraisal. The findings from case reports using mono-JWXYS and pharmacological studies were summarized in tables.

**Results:**

Thirteen RCTs with 979 participants were included. The majority of the included studies were assessed as high risk of bias in one ROB domain. Compared with ATDs, JWXYS plus ATDs resulted in lower free triiodothyronine (FT3) (MD = -1.31 pmol/L, 95% CI [-1.85, -0.76]; low-certainty), lower free thyroxine (MD = -3.24 pmol/L, 95% CI [-5.06, -1.42]; low-certainty), higher thyroid stimulating hormone (MD = 0.42 mIU/L, 95% CI [0.26, 0.59]; low-certainty), higher effectiveness rate of traditional Chinese medicine syndrome (RR = 1.28, 95% CI [1.08, 1.52]; low-certainty), lower goiter score (MD = -0.66, 95% CI [-1.04, -0.29]; very low-certainty), lower thyrotrophin receptor antibody (SMD = -0.44, 95% CI [-0.73, -0.16]; low-certainty) and fewer adverse events (AEs) (RR = 0.34, 95% CI [0.18, 0.67]; moderate-certainty). Compared with regular dosage of ATDs, JWXYS plus half-dose ATDs resulted in fewer AEs (RR = 0.24, 95% CI [0.10, 0.59]; low-certainty). Compared with ATDs in 1 trial, JWXYS resulted in higher FT3, lower goiter score and fewer AEs. Three case reports showed that the reasons patients sought TCM-only treatment include severe AEs and multiple relapses. Three pharmacological studies demonstrated that JWXYS restored Th17/Treg balance, lowered deiodinases activity, regulated thyroid cell proliferation and apoptosis, and alleviated liver oxidative stress in mouse or rat models.

**Conclusion:**

JWXYS may enhance the effectiveness of ATDs for hyperthyroidism, particularly in relieving symptoms and reducing AEs. Mono-JWXYS is not recommended except in patients intolerant to ATDs. The findings should be interpreted with caution due to overall high risk of bias. Further pharmacological studies with more reliable models are needed.

**Systematic review registration:**

https://www.crd.york.ac.uk/prospero/, identifier CRD42023394923.

## 1 Introduction

Hyperthyroidism is characterized by excessive production and secretion of thyroid hormones (THs) by the hyperactive thyroid gland (1), which is a significant public health concern worldwide. Excessive THs affect almost all tissues and organ systems, and some of the most profound effects occur within the cardiovascular system (2). Thyrotoxicosis is associated with a range of complications, including osteoporosis, atrial fibrillation, embolic events, periodic paralysis, neuropsychiatric symptoms, abnormalities in the reproductive system, and, rarely, cardiovascular collapse and death (3). Atrial fibrillation-related embolic stroke secondary to hyperthyroidism is significantly more common than from non-thyroidal causes (4). Patients with hyperthyroidism have an increased risk of all-cause mortality, with heart failure being the leading cause of increased cardiovascular mortality (5). The prevalence of hyperthyroidism ranges from 1.2% to 1.6% and is reported to be on the rise (6). The most common cause of hyperthyroidism in iodine-sufficient areas is Graves’ disease (GD), accounting for 75%, followed by toxic nodular goiter (7). GD is an organ-specific autoimmune disease directly caused by thyrotrophin receptor antibody (TRAb) that binds to the thyrotropin receptor (TSHR), subsequently inducing the synthesis and release of THs, and diffuse enlargement of the thyroid gland.

The treatment of hyperthyroidism involves both symptom relief and a definitive cure (8), aiming to improve health-related quality of life (HRQoL) and reduce morbidity and mortality. Three potent classical approaches are offered to treat hyperthyroidism: blocking THs production with antithyroid drugs (ATDs), destroying the thyroid tissue with radioactive iodine (RAI), or surgical removal (thyroidectomy). However, it has been reported in patients with GD or toxic nodular goiter that severe disease-specific and generic HRQoL impairments persist up to 6 months after treatment of ATDs, RAI or thyroidectomy (9, 10), even in euthyroid patients. These impairments can last for many years after diagnosis, involving goiter symptoms, hyperthyroid symptoms, tiredness, cognitive complaints, anxiety, emotional susceptibility and impaired quality of social and daily life (9, 11). In recent decades, ATDs have been the first-line patient-preferred treatment worldwide, considering complications, financial cost, and impaired quality of life induced by RAI or thyroidectomy (11–13). ATDs are typically given for 18 - 24 months, but have an extremely high risk of relapse after withdrawal, approximately 50% at one year after discontinuation (14). In addition, ATDs may cause some side effects, with mild skin allergic reactions, agranulocytosis and hepatic dysfunction being the most common (15). Sometimes, when the side effects are too severe, ATDs need to be discontinued. The evidence-based clinical guideline for the management of thyrotoxicosis published by the American Thyroid Association in 2016 state that there is insufficient evidence to support the recommendation of alternative therapies alone for the treatment of hyperthyroidism. Therefore, there is a need to explore complementary and alternative therapies to control hyperthyroidism and relieve symptoms.

In China and some other countries, Chinese herbal medicines (CHMs) are widely accepted by patients and clinicians in the treatment of hyperthyroidism (16–18). The mechanism of CHMs in treating hyperthyroidism has also been widely explored (19). In a population-based study in Taiwan, CHMs were reported to be used in 73% of the patients with hyperthyroidism, and Jiawei-Xiaoyao-San (JWXYS) was the most commonly chosen prescription for hyperthyroidism (20).​ Many previous clinical studies have shown that the combination of JWXYS with ATDs in the treatment of hyperthyroidism can improve clinical effectiveness or reduce the dose and side effects of ATDs, and in particular, can significantly relieve symptoms (21, 22). Furthermore, some case reports indicate that JWXYS may be a possible alternative treatment for ATDs in the presence of contraindications (16, 23, 24). However, the clinical evidence and mechanisms have not been systematically reviewed. In order to provide decision guidance for clinicians, we conducted this systematic review and meta-analysis of existing randomized controlled trials (RCTs) of JWXYS for the treatment of hyperthyroidism, and case reports were also considered to assess the application condition. In addition, we conducted a review of the pharmacological mechanisms of JWXYS in the treatment of hyperthyroidism.

## 2 Methods

This systematic review was conducted referring to the Cochrane Handbook for Systematic Reviews of Interventions (25) and is reported according to the Preferred Reporting Items for Systematic Review (PRISMA) 2020 (26) (**Supplementary Material S1**). The protocol was registered in PROSPERO, and the registration number is CRD42023394923.

### 2.1 Eligibility criteria

#### 2.1.1 Eligibility criteria of clinical trials for meta-analysis

(1) Type of Study: RCTs were selected for meta-analysis. Studies published in journals with only one author were identified as non-RCTs and excluded, even when randomization were mentioned.

(2) Participants: Participants diagnosed with hyperthyroidism by explicit diagnostic criteria referring to guidelines or textbooks were included. There were no restrictions on age, race, gender or region. Participants with co-morbidities or other common conditions that had little or no effect on thyroid function were also eligible. Trials in which participants’ thyroid functions were in stable state were excluded.

(3) Interventions: The intervention groups were treated with JWXYS plus conventional therapies or alone, regardless of modification, dosage form, or administration route. Considering the large storage of thyroid hormone in thyroid follicular colloid, the intervention duration should be at least 4 weeks.

(4) Comparisons: Studies in which the control groups were treated with ATDs for hyperthyroidism and without any TCM therapies were included.

(5) Outcome measures: The primary outcomes were three thyroid functions, including free triiodothyronine (FT3), free thyroxine (FT4) and thyroid stimulating hormone (TSH). The secondary outcomes included the effectiveness rate of TCM syndrome, the TCM syndrome score, the degree of goiter, serum concentration of TRAb, relapse during follow-up, along with occurrence of adverse events (AEs, including skin allergic reactions, agranulocytosis and hepatic dysfunction). Trials that reported at least one primary outcome were included.

#### 2.1.2 Eligibility criteria of case reports

Case reports using JWXYS alone in the treatment of hyperthyroidism were included for qualitative study.

#### 2.1.3 Eligibility criteria of pharmacological studies

Original articles investigating the pharmacological mechanism of JWXYS in controlling hyperthyroidism were included. The disease model used should be consistent with the pathological characteristics of hyperthyroidism. If medication other than JWXYS were used, both the treatment and control groups must be administered simultaneously. THs should be measured as the primary outcome, and JWXYS should be proved effective in controlling hyperthyroidism.

### 2.2 Information sources and search strategy

Eight electronic databases and three clinical trial registries were searched from their inception to May 2, 2023, including PubMed, Cochrane Library, Embase, Web of Science, China National Knowledge Infrastructure (CNKI), Chongqing Chinese Science and Technology Journal Database (VIP), China BioMedical Literature Service System (SinoMed), Wanfang Database, World Health Organization International Clinical Trials Registry Platform (https://www.who.int/clinical-trials-registry-platform), ClinicalTrials.gov, and the Chinese Clinical Trial Registry (ChiCTR, http://www.chictr.org.cn/index.aspx). There were no language and publication restrictions. The references of reviews or clinical trials were searched as supplements. Search terms, such as “hyperthyroidism”, “Graves’ disease”, “Jiawei-Xiaoyao-San”, and “Danzhi-Xiaoyao-San” were identified according to previous systematic reviews, clinical practice guidelines, MeSH terms and Emtree. Detailed search strategies are presented in **Supplementary Material S2** (**Table 1**).

**Table 1:** Summary of the characteristics in each included RCTs.

Author (Year)	Funding sources	classification	Comorbidities	Sample Size (M/F)		Age [years] (mean ± SD)		Duration of Disease [months]		Treatment Duration	Interventions			Outcome measure (s)	TCM syndrome differentiation
				I	C	I	C	I	C		I (Constituent herbs [g] and Administration)	C (Dosage [mg] and Administration)	Basic treatment		
Wang SL (2011)	NR	GD	NR	30 (8/22)	30 (9/21)	39.27 ± 11.45	39.53 ± 11.94	NR	NR	2m	Modified JWXYS (209, bid) + Halved C	Thiamazole (10*, tid)	NR	1, 2, 3, 5, 6, 8	Hyperactivity of heart-liver fire
Tang YL (2012)	NR	GD	Hashimoto’s thyroiditis	29 (5/24)	26 (4/22)	38.57 ± 9.84	37.33 ± 9.19	NR	NR	4w	Modified JWXYS (183, bid)	Thiamazole (5, tid)	BT, propranolol (10mg, tid)	1, 2, 3, 4, 5, 6, 8	Liver depression with fire and spleen deficiency
Liu SY et al. (2012 + 2012 + 2016)	Government	GD	NR	36 (5/31)	36 (7/29)	30.08 ± 11.78	33.47 ± 9.56	NR	NR	12w	Modified JWXYS (226, tid) + C	Thiamazole (10*, tid)	BT	1, 2, 3, 4, 5, 6, 8	Liver depression inducing fire
Huang FX et al. (2013)	NR	NR	Hepatic dysfunction	35 (10/25)	35 (11/24)	35.4 ± 12.6	36.3 ± 14.1	57.6 ± 7.2	40.8 ± 8.4	1m	Modified JWXYS (154, bid) + Halved C	Thiamazole (10, tid)	BT, propranolol (10mg, tid)	1, 2, 3, 8	NR
Guo J (2015)	NR	GD	NR	30 (11/19)	30 (9/21)	37.97 ± 9.33	37.67 ± 9.59	8.76 ± 6.28	8.48 ± 6.38	3m	Modified JWXYS (73, bid) + C	Thiamazole (10-20/d*)	BT	1, 2, 3, 4, 8	Prosperity of liver fire
Qiu ZQ (2015)	NR	NR	Heart disease	56 (25/31)	52 (22/30)	46.29 ± 11.27	44.72 ± 10.02	56.04 ± 14.76	57.72 ± 15.72	8w	Modified JWXYS (156, bid) + C	Thiamazole (20/d) or Propylthiouracil (200-300/d); treatment for heart disease^#^	NR	1, 2, 3, 8	NR
Li MY (2016)	NR	NR	NR	36 (5/31)	36 (4/32)	range:18~70		<12		4w	Modified JWXYS (226, tid) + C	Thiamazole (10*, tid)	BT	1, 2, 3, 4, 5	Liver depression inducing fire
Wu MY (2017)	NR	GD	NR	30 (7/23)	30 (9/21)	31.1 ± 10.2	33.6 ± 8.98	5.53 ± 3.2	5.33 ± 2.62	6m	Modified JWXYS (205, bid) + C	Thiamazole (10*, tid)	BT	1, 2, 3, 5, 6, 8	Liver depression inducing fire
Zhang LL (2017)	NR	GD	Hepatic dysfunction	50 (26/24)	50 (28/22)	44.63 ± 16.75	43.85 ± 15.29	10.44 ± 18.72	9.72 ± 18.96	12w	Modified JWXYS (226, bid) + C	Thiamazole (10*, tid); diammonium glycyrrhizinate enteric-coated capsule (150, tid)	BT	1, 2, 3, 7	Liver depression inducing fire
Li JN (2018)	NR	GD	NR	36 (4/32)	36 (7/29)	33.89 ± 11.22	36.19 ± 13.11	12.97 ± 9.09	11.75 ± 8.84	12w	Modified JWXYS (195, tid) + C	Thiamazole (30-45/d*)	NR	1, 2, 3, 4, 5, 6, 8	Prosperity of liver fire
Tian LY et al. (2020)	Government	NR	NR	49 (15/34)	49 (13/36)	39.6 ± 7.4	37.8 ± 7.2	10.4 ± 3.5	12.3 ± 2.6	4w	Modified JWXYS (189, bid) + C	Thiamazole (10-40, tid)	NR	1, 2, 3, 4, 6, 7, 8	Liver depression inducing fire
Shi LJ et al. (2020)	Government	NR	NR	45 (9/36)	45 (7/38)	36.16 ± 3.13	35.58 ± 3.23	5.36 ± 1.02	5.44 ± 0.97	3m	Modified JWXYS (122, bid) + C	Thiamazole (10*, tid)	NR	1, 2, 3, 4	Liver depression and spleen deficiency
Gao R et al. (2021)	Government	GD	NR	60 (0/60)	60 (0/60)	36.24 ± 10.32	38.35 ± 10.73	13.68 ± 5.04	14.88 ± 3.84	3m	Modified JWXYS (150, bid) + C	Thiamazole (10*, bid)	BT	1, 2, 8	Prosperity of liver fire

I: intervention group; C: control group; M: male; F: female; m: months; w: weeks; d: day; NR: not reported; *: Reduce the dosage gradually according to thyroid functions; BT: basic treatments, including low iodine diet, adequate rest and nutrition, appropriate exercise and emotion regulation et al.; ^#^: propranolol 20-30/d for tachycardia or atrial fibrillation, diuretics, vasodilators and digoxin or low-dose propranolol for heart failure; tid: 3 times a day; bid: 2 times a day. Outcome measure (s): 1: FT3, 2: FT4, 3: TSH, 4: effectiveness rate of TCM syndrome, 5: TCM syndrome score, 6: Goiter score, 7: TRAB, 8: Adverse events.

### 2.3 Selection and data collection process

The screening was conducted independently by two authors (GW and MYX) according to the eligibility criteria. Retrieval results were imported into NoteExpress (Beijing Aegean Software company, v3.8.0.9492) and automatic duplicate removal was conducted. The first screening was conducted according to the titles and abstracts of all potential studies, and then the full texts were obtained for the second screening. Two authors (RTZ and WXM) independently extracted data from all eligible trials. A data extraction spreadsheet was designed and modified for this review. If the required data were not available in the trial, further information was sought by contacting the original researchers. Data from trials published in duplicate were extracted only once. Discussions and a third reviewer’s (ZLL) proposal were required if there was any discrepancy.

### 2.4 Data items included

#### 2.4.1 Data from RCTs

Basic information of study, characteristics of participants, and details of interventions were extracted as follows: the first author’s name, publication year, funding source, sample size, age and gender of participants, duration of disease, diagnostic criteria, treatments with their dosage and administration route from all groups, dosage of each ingredient in JWXYS and modification, duration of intervention, and quality assessment.

The outcome measurements were extracted emphatically, including FT3, FT4, TSH, effectiveness rate of TCM syndrome, TCM syndrome score, TRAb, goier score, relapse rate and AEs. The criterion of effectiveness rate of TCM syndrome should be in compliance with the Chinese Medicine Clinical Research Guidelines (CMCRG) (27) and was defined as a significant amelioration or disappearance of clinical symptoms and signs of TCM syndrome, and a reduction in syndrome score of more than 70%. In all studies included in this review, the degree of goiter was divided into three grades: Grade I: The thyroid gland is not swollen in appearance, but can be palpated; Grade II: Goiter is visible and palpable, but the goiter does not extend beyond the outer margin of the sternocleidomastoid muscle; Grade III: The goiter extends beyond the outer margin of the sternocleidomastoid muscle. The degree of goiter was denoted by goiter score, and each grade accumulates two points. Data from all post-intervention time points were extracted to find the most reported time points.

#### 2.4.2 Data from case reports

Basic information of study, characteristics of participants, details of interventions and outcomes were included as follows: first author’s name, publication year, age and gender of participants, medical history, duration of disease, diagnosis, treatments with dosage and administration route, dosage of each ingredient in JWXYS and modification, duration of intervention, and changes in clinical manifestation and laboratory tests.

#### 2.4.3 Data from mechanism studies

The first author’s name, publication year, study model, treatments of the experimental group and control group, and changes in outcomes for efficacy evaluation and mechanism research were all extracted.

### 2.5 Study risk of bias assessment

Two authors (RTZ and WXM) used the Cochrane Risk-of-Bias (ROB) 1.0 tool in Review Manager (RevMan) 5.4 software to evaluate the quality of the included RCTs with reference to the following aspects: (a) random sequence generation (selection bias), (b) allocation concealment (selection bias), (c) blinding of participants and personnel (performance bias), (d) blinding of outcome assessment (detection bias), (e) incomplete outcome data (attrition bias), (f) selective reporting (reporting bias), (g) other bias. Each aspect was judged as low, unclear or high. Discrepancies were resolved by discussion or involving a third author (ZLL). For case reports, we used the Joanna Briggs Institute (JBI) tool 2020 (28) for critical appraisal.

### 2.6 Effect measures

For RCTs, data analyses were performed using RevMan 5.4. Dichotomous variables were presented as risk ratio (RR) with 95% confidence interval (CI). Continuous variables were presented as weighted mean difference (MD) with 95% CI if the measurement units of outcomes were the same in different trials, and the standardized mean difference (SMD) was used when the measurement units of outcomes differed from one another.

### 2.7 Synthesis methods

Trial characteristics were summarized in tables by Microsoft excel 2021. For RCTs, the meta-analysis was performed using RevMan 5.4. Forest plots were used to describe the results. For the comparison of different interventions and outcomes, the results were synthesized respectively. We intended to pool the collected data at similar time points, with data from one trial being synthesized only once. If the standard deviation was not reported by means, it was calculated from the information reported (29). If the required data were unavailable in the trial and we failed to contact the original researchers, we only used the available data in the analyses.

The heterogeneity test was performed by chi-square test and presented as the value of I^2^ statistics. According to the Cochrane handbook, more than 25%, 50%, and 75% values were considered as mild, moderate, and severe heterogeneity, respectively (25). The fixed-effects model (FEM) was applied when I^2^ < 50%; otherwise, the random-effects model (REM) was applied. The Mantel-Haenszel method was used for meta-analyses of dichotomous outcomes. The inverse-variance method was used for meta-analyses of continuous outcomes. The subgroup analysis was conducted to explore any factors that might explain the heterogeneity. Overall effects with a p-value below 0.05 were considered statistically significant.

Sensitivity analysis was performed using STATA 14.0 software for the primary outcome to determine whether the conclusion was robust. When severe heterogeneity existed, sensitivity analysis was conducted for potential sources of heterogeneity.

### 2.8 Publication bias and certainty assessment

Funnel plot and Egger’s test (30) was performed to assess potential publication bias in a single meta-analysis involving ten or more trials. Two authors (RTZ and WXM) conducted the certainty assessment of each outcome using the GRADEpro Guideline Development Tool (GRADEpro GDT) developed by the Grading of Recommendations, Assessment, Development, and Evaluation (GRADE) working team. Discrepancies were resolved by discussion or involving a third author (ZLL).

## 3 Results

### 3.1 Study selection

**Figure 1:**
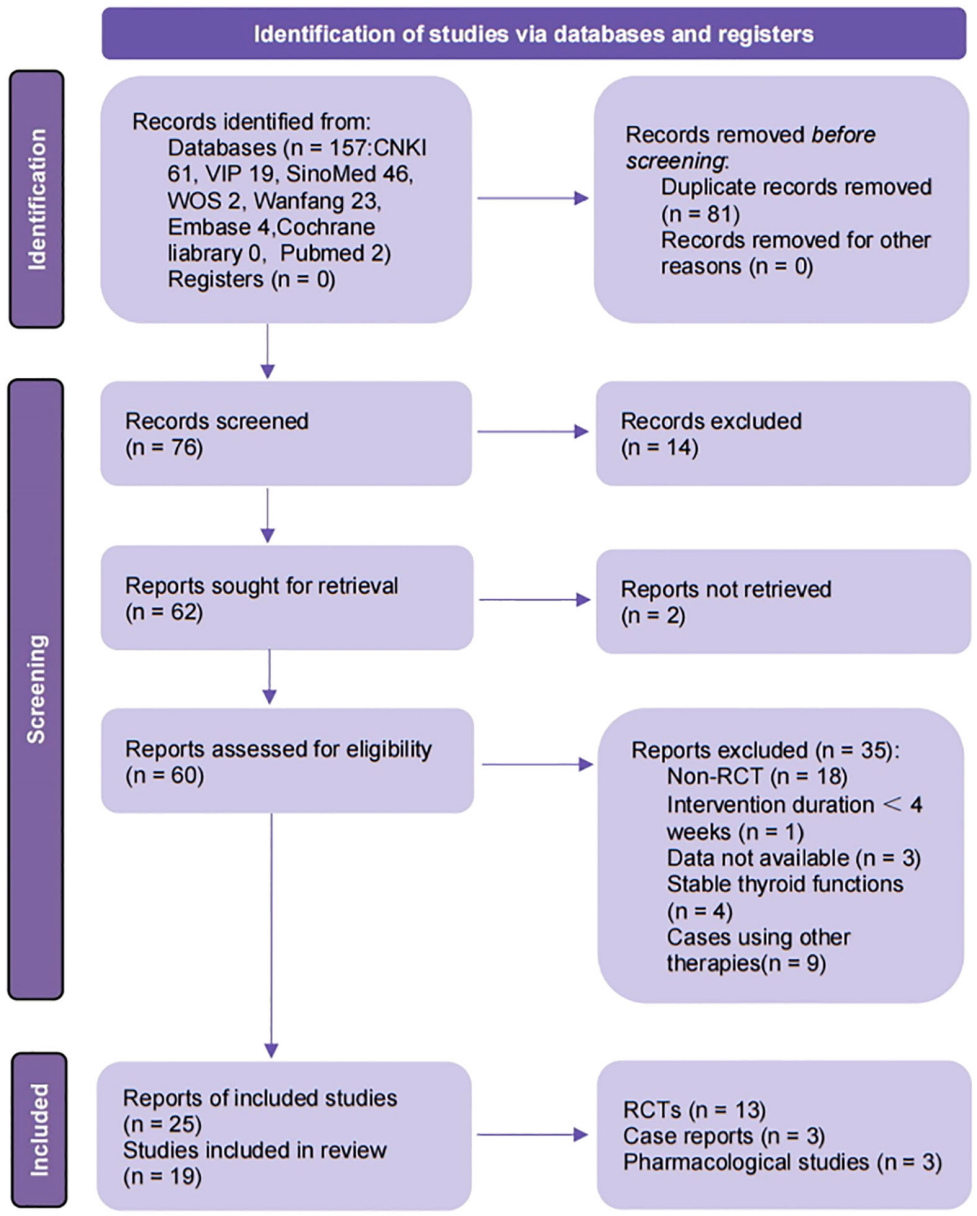
Flow diagram of study selection process. CNKI, China National Knowledge Infrastructure; VIP, Chongqing Chinese Science and Technology Journal Database; SinoMed, China BioMedical Literature Service System; ICTRP, the WHO International Clinical Trials Registry Platform; n, number; RCT, randomized controlled trails. Template for the flow diagram was provided by PRISMA.

From 8 databases and 3 clinical trial registries, 157 records were identified. After removing 81 duplicates, 14 records irrelevant to this review were excluded, leaving 62 records for full-text screening. Of these, 60 reports were obtained in full text. For RCTs, 18, 1, 3, and 4 reports were excluded due to lack of randomization, duration of intervention less than 4 weeks, no available data, and stable thyroid function of participants, respectively. Nine case reports were excluded due to the intervention including other therapies. Three reports contained data from the same study. Finally, 25 reports (15 reports including 13 RCTs, 3 case reports including 3 cases and 7 reports including 3 experiments for mechanism research) were assessed to meet the eligibility criteria. The detailed screening process is shown in the PRISMA flow diagram (**Figure 1**) (26).

### 3.2 Study characteristics of included RCTs

In 13 RCTs from 15 reports, a total of 979 participants were enrolled, age ranged from 16 to 76 years old, with 485 in the control group and 494 in the intervention group. The data in three reports (31–33) came from the same trial. In 9 trials, hyperthyroidism in patients was explicitly described as being caused by GD, which was not reported in the other trials. Treatment duration ranged from 4 weeks to 6 months, with the most common being 12 weeks (considered equivalent to 3 months). Therefore, when outcomes at multiple time points were reported, we extracted outcomes at or closest to 12 weeks. In terms of intervention measures, 10 trials compared JWXYS plus ATDs with the same dose of ATDs (22, 24, 31–43), 2 trials compared JWXYS plus half-dose ATDs with normal dose ATDs (21, 43), and only one trial compared JWXYS with ATDs (44). Two trials reported hepatic dysfunction (21, 32), one reported heart disease (40) and one reported Hashimoto’s thyroiditis (44) as comorbidity. JWXYS was administered as a modified decoction in all trials. The most common TCM syndromes are “Liver depression inducing fire” (in 5 trials), “Prosperity of liver fire” (in 3 trials), “Liver depression with fire and spleen deficiency” (in 2 trials) and “Hyperactivity of heart-liver fire” (in 1 trial). The detailed characteristics of the included trials are shown in **Table 1**. The detailed components of the prescriptions used in each study are summarized in the **Supplementary Material S2** (**Table 2**).

**Table 2:** Effectiveness and safety of JWXYS for hyperthyroidism based on 13 randomized controlled trials.

Outcomes	No. of studies	No. of participants	I² (%), Effect model (R/F)	Type of effect size	Effect size [95%CI]	Quality of evidence(GRADE)
JWXYS + ATD versus ATD						
FT3 (pmol/L)	10	794	89, R	MD	-1.31 [-1.85, -0.76]	Low ^a,e^
FT4 (pmol/L)	10	794	97, R	MD	-3.24 [-5.06, -1.42]	
TSH (mIU/L)	8	672	86, R	MD	0.42 [0.26, 0.59]	
Effectiveness rate of TCM syndrome	6	464	0, F	RR	1.28 [1.08, 1.52]	Low ^b^
TCM syndrome score	5	336	94, R	SMD	-1.62 [-2.68, -0.56]	Very low ^b,e^
Goiter score	5	382	72, R	MD	-0.66 [-1.04, -0.29]	Very low ^b,e^
TRAb	2	198	0, F	SMD	-0.44 [-0.73, -0.16]	Low ^a,c^
Adverse events	7	534	0, F	RR	0.34 [0.18, 0.67]	Moderate ^a,d,f^
JWXYS + L-ATD versus ATD						
FT3 (pmol/L)	2	130	55, R	MD	0.47 [-0.75, 1.68]	Very low ^a,c,e^
FT4 (pmol/L)	2	130	57, R	MD	-1.63 [-4.81, 1.56]	
TSH (mIU/L)	2	130	78, R	MD	-0.02 [-0.50, 0.46]	
TCM syndrome score	1	60	NA, R	MD	-2.87 [-5.01, -0.73]	Very low ^b,c^
Goiter score	1	60	NA, R	MD	-0.07 [-0.86, 0.72]	Very low ^b,c^
Adverse events	2	130	0, F	RR	0.24 [0.10, 0.59]	Low ^a,c,d,f^
JWXYS versus ATD						
FT3 (pmol/L)	1	55	NA, R	MD	1.53 [0.50, 2.56]	Low ^a,c^
FT4 (pmol/L)	1	55	NA, R	MD	3.07 [-0.04, 6.18]	
TSH (mIU/L)	1	55	NA, R	MD	-0.04 [-0.13, 0.05]	
Effectiveness rate of TCM syndrome	1	55	NA, F	RR	4.50 [0.23, 89.62]	Very low ^b,c^
TCM syndrome score	1	55	NA, R	MD	-3.16 [-5.26, -1.06]	
Goiter score	1	55	NA, R	MD	-1.17 [-2.17, -0.17]	Very low ^b,c^
Adverse events	1	55	NA, F	RR	0.05 [0.00, 0.78]	Low ^a,c,d,f^

JWXYS: Jiawei-Xiaoyao-San; ATD: antithyroid drug; L-ATD: half-dose ATD; NA: not applicable; R: randomized-effects model; F: fixed-effects model; RR: risk ratio; SMD: standardized mean difference; MD: weighted mean difference; ^a^: serious risk of bias, including a lack of description of randomization and blinding; ^b^: extremely serious risk of bias, including a lack of description of randomization and blinding and the subjectivity of outcome measurement; ^c^: serious imprecision due to insufficient sample size; ^d^: serious imprecision due to the infrequency of events; ^e^: serious inconsistency due to obvious heterogeneity; ^f^: upgrade due to large effect size.

### 3.3 Risk of bias in included RCTs

**Figure 2:**
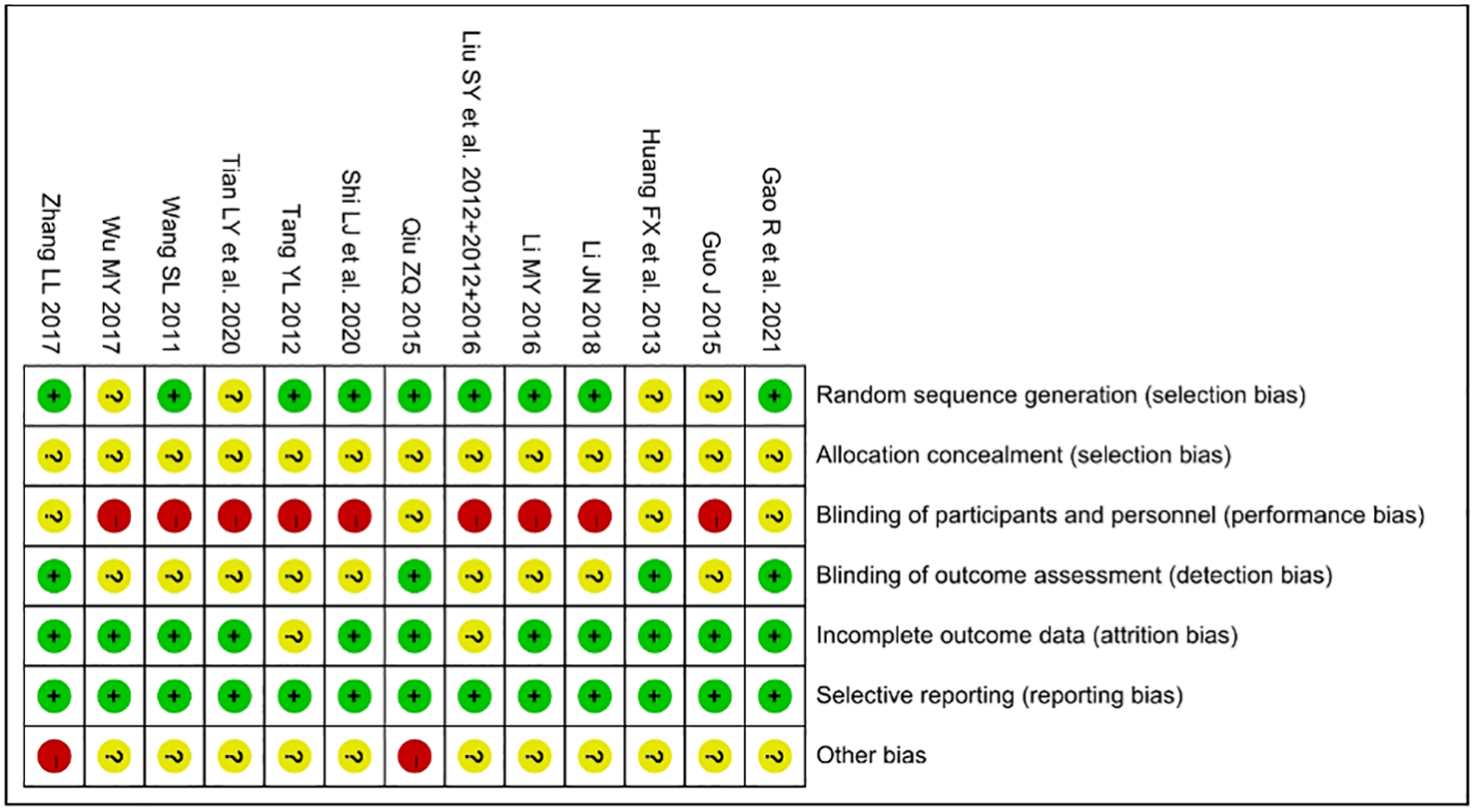
Risk of bias summary of 13 included RCTs.

The risk of bias on primary outcomes in 13 RCTs was summarized in **Figure 2**. Nine trials reported the use of random number table as the random sequence generation method, which was considered to have a “low risk of bias”. Five trials just mentioned “randomization” without detailed methods (21, 37, 40, 41, 44) and were considered to have an “unclear risk of bias” in random sequence generation. None of the trials mentioned the details of allocation concealment of randomization methods and implementation of blinding. All trials were considered to have an “unclear risk of bias” in allocation concealment. The majority of the trials were considered to have a “high risk of bias” in performance bias due to apparently different intervention without placebo. Four trials (21, 22, 32, 40) were considered to have an “unclear risk of bias” in performance bias and a “low risk of bias” in detection bias because they only reported physiological indicator as outcomes. In the aspect of “incomplete outcome data”, two trials (31–33, 44) that did not report the cause of the missing data were considered to have an “unclear risk of bias”. In two trials (32, 40), the proportion of male and female participants did not correspond to the clinical ratio of men to women, therefore, these trials were considered to have a “high risk of bias” in other bias.

### 3.4 Effectiveness of interventions

#### 3.4.1 Primary outcomes

The meta-analyses showed that compared with ATDs, the combination of JWXYS and ATDs resulted in lower FT3 (MD = -1.31 pmol/L, 95% CI: -1.85 to -0.76; 10 trials, 794 participants), lower FT4 (MD = -3.24 pmol/L, 95% CI: -5.06 to -1.42; 10 trials, 794 participants) and higher TSH (MD = 0.42 mIU/L, 95% CI: 0.26 to 0.59; 8 trials, 672 participants).

For the comparison between regular-dose ATDs and JWXYS plus half-dose ATDs (2 trials, 130 participants), the meta-analyses showed no significant difference on the previous thyroid functions.

One trial showed that compared with ATDs, mono-JWXYS resulted in higher FT3 (MD = 1.53 pmol/L, 95% CI: 0.50 to 2.56; 55 participants), higher FT4 (MD = 3.07 pmol/L, 95% CI: -0.04 to 6.18; 55 participants) and similar TSH.

**Figure 3:**
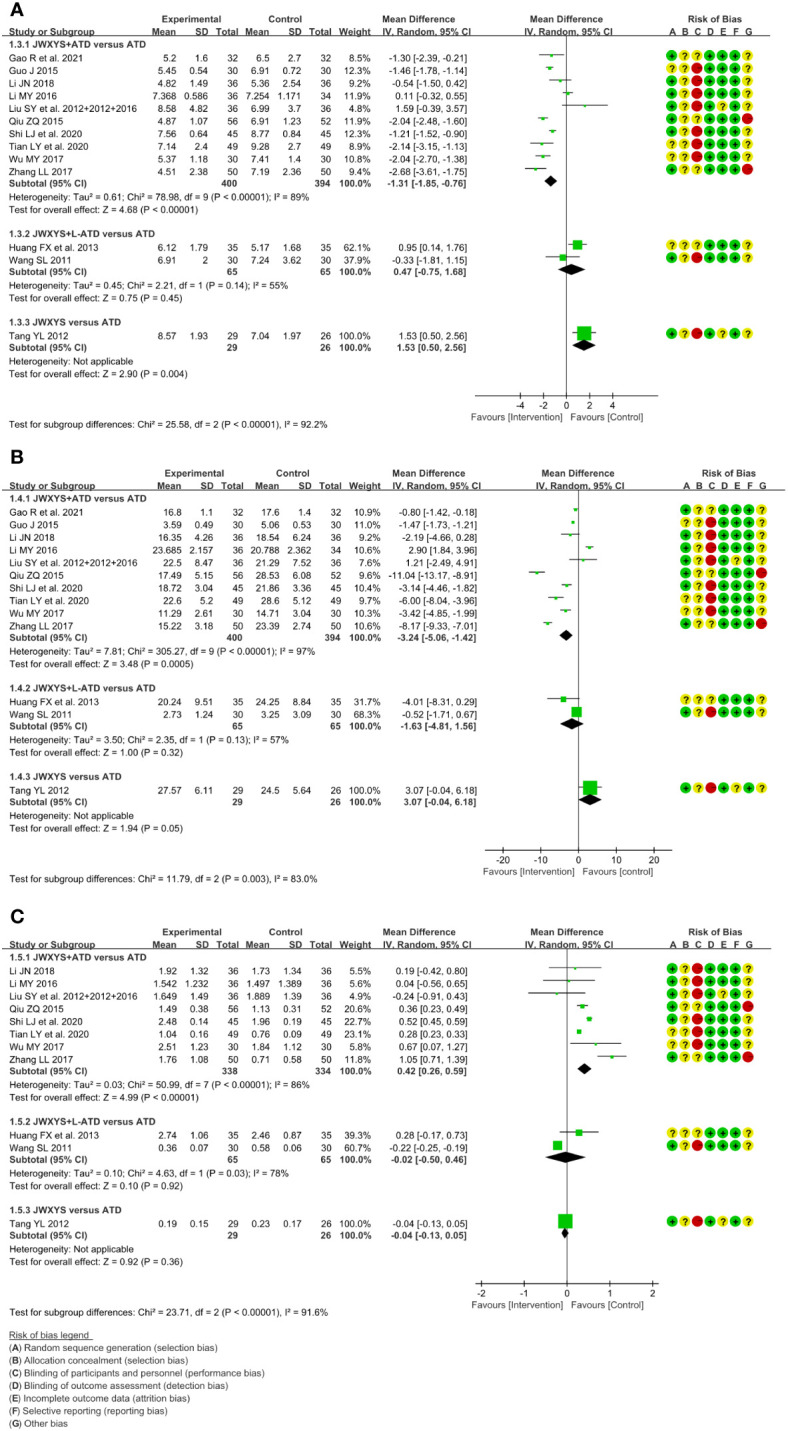
Forest plots of the effectiveness comparison on thyroid functions. **(A)**: Free triiodothyronine, **(B)**: Free thyroxine, **(C)**: Thyroid stimulating hormone. JWXYS, Jiawei-Xiaoyao-San; ATD, antithyroid drug; L-ATD, half-dose antithyroid drug.

The heterogeneity among different comparisons was significantly high (*P* < 0.00001) in each outcome. The detailed results are summarized in **Table 2** and shown as forest plots in **Figure 3**.

#### 3.4.2 Secondary outcomes

##### 3.4.2.1 TCM syndrome

The meta-analyses showed that compared with ATDs alone, the combination of JWXYS and ATDs resulted in higher effectiveness rate of TCM syndrome (RR = 1.28, 95% CI: 1.08 to 1.52; 6 trials, 464 participants) and lower TCM syndrome score (SMD = -1.62, 95% CI: -2.68 to -0.56; 5 trials, 336 participants).

One trial showed that compared with regular-dosage of ATDs, the combination of JWXYS and half-dose ATDs resulted in lower TCM syndrome score (MD = -2.87, 95% CI: -5.01 to -0.73; 60 participants).

One trial showed that compared with ATDs, mono-JWXYS resulted in higher effectiveness rate of TCM syndrome (RR = 4.50, 95% CI: 0.23 to 89.62; 55 participants), lower TCM syndrome score (MD = -3.16, 95% CI: -5.26 to -1.06; 55 participants).

**Figure 4:**
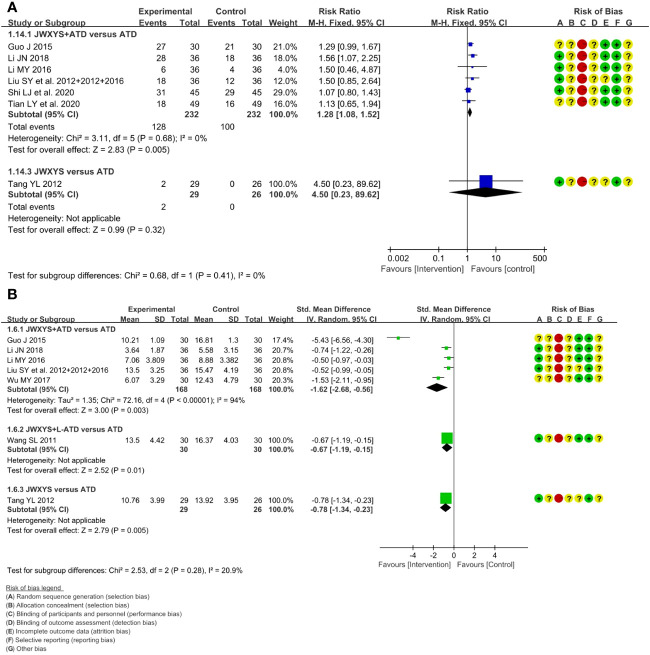
Forest plots of the effectiveness comparison on traditional Chinese medicine (TCM) syndrome. **(A)**: Effectiveness rate of TCM syndrome, **(B)**: TCM syndrome score. JWXYS, Jiawei-Xiaoyao-San; ATD, antithyroid drug; L-ATD, half-dose antithyroid drug.

The heterogeneity among different comparisons was not significant. The detailed results are summarized in **Table 2** and shown as forest plots in **Figure 4**. It’s worth noting that the symptoms and signs included in various studies were not exactly the same, mainly including irritability, intolerance to heat, palpitation, hyperphagia, tremor, emaciation, fatigue, sweating, insomnia, etc.

##### 3.4.2.2 Goiter score

The meta-analysis showed that compared with ATDs alone, the combination of JWXYS and ATDs resulted in lower goiter score (MD = -0.66, 95% CI: -1.04 to -0.29; 5 trials, 362 participants).

One trial showed that compared with regular-dosage of ATDs, the combination of JWXYS and half-dose ATDs resulted in similar goiter score (MD = -0.07, 95% CI: -0.86 to 0.72; 60 participants).

One trial showed that compared with ATDs, mono-JWXYS resulted in lower goiter score (MD = -1.17, 95% CI: -2.17 to -0.17; 55 participants).

**Figure 5:**
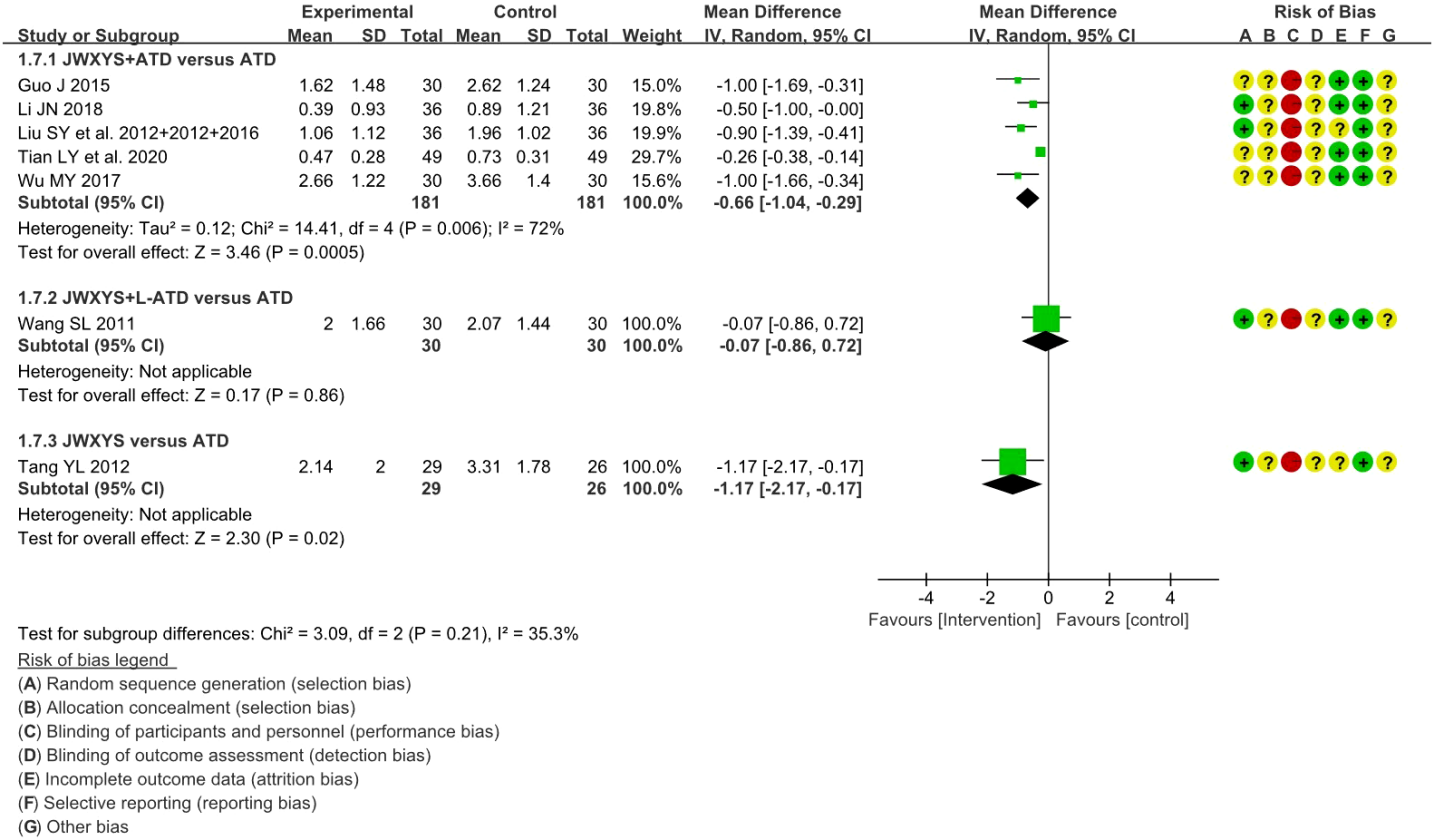
Forest plots of the effectiveness comparison on goiter score. JWXYS, Jiawei-Xiaoyao-San; ATD, antithyroid drug; L-ATD, half-dose antithyroid drug.

The detailed results are summarized in **Table 2** and shown as forest plots in **Figure 5**.

##### 3.4.2.3 TRAb

**Figure 6:**
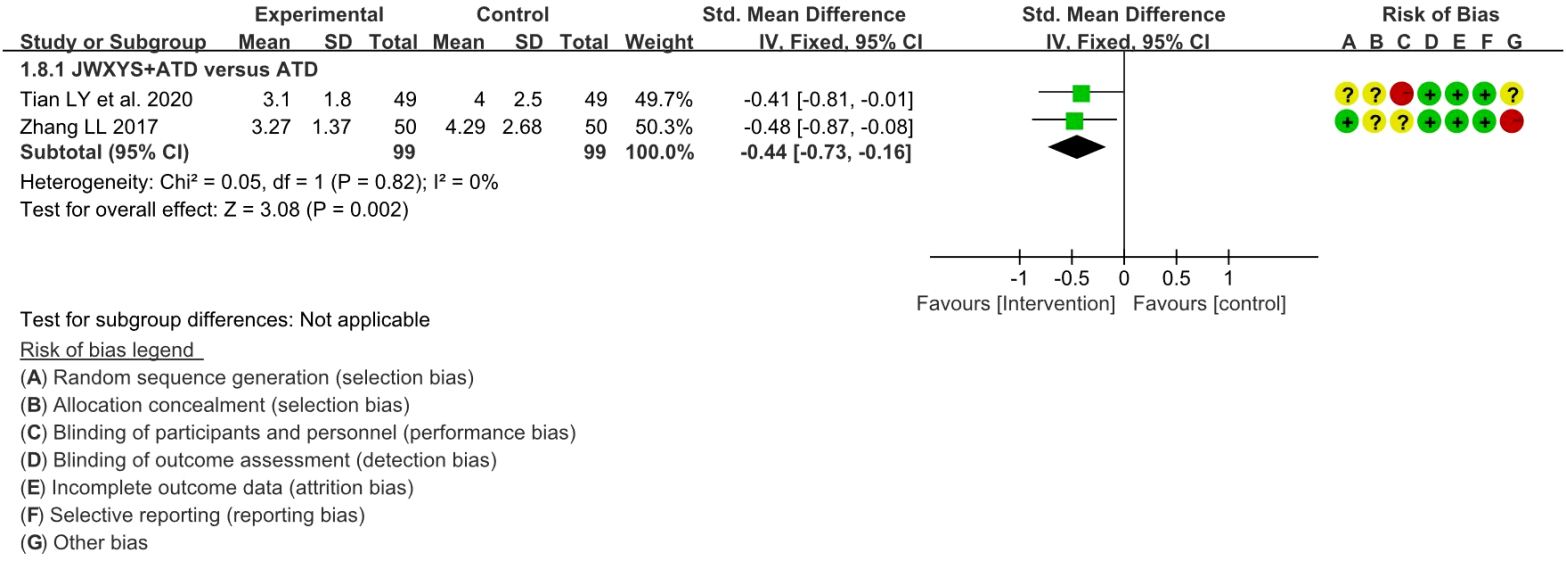
Forest plot of the effectiveness comparison on TRAb. JWXYS, Jiawei-Xiaoyao-San; ATD, antithyroid drug.

The meta-analyses showed that compared with ATDs alone, the combination of JWXYS and ATDs resulted in lower TRAb (SMD = -0.44, 95% CI: -0.73 to -0.16; 2 trials, 198 participants). TRAb was not reported in other comparisons. The detailed results are summarized in **Table 2** and shown as forest plot in **Figure 6**.

##### 3.4.2.4 Recurrence rate

No trial reported the recurrence rate at follow-up.

##### 3.4.2.5 Adverse events

**Figure 7:**
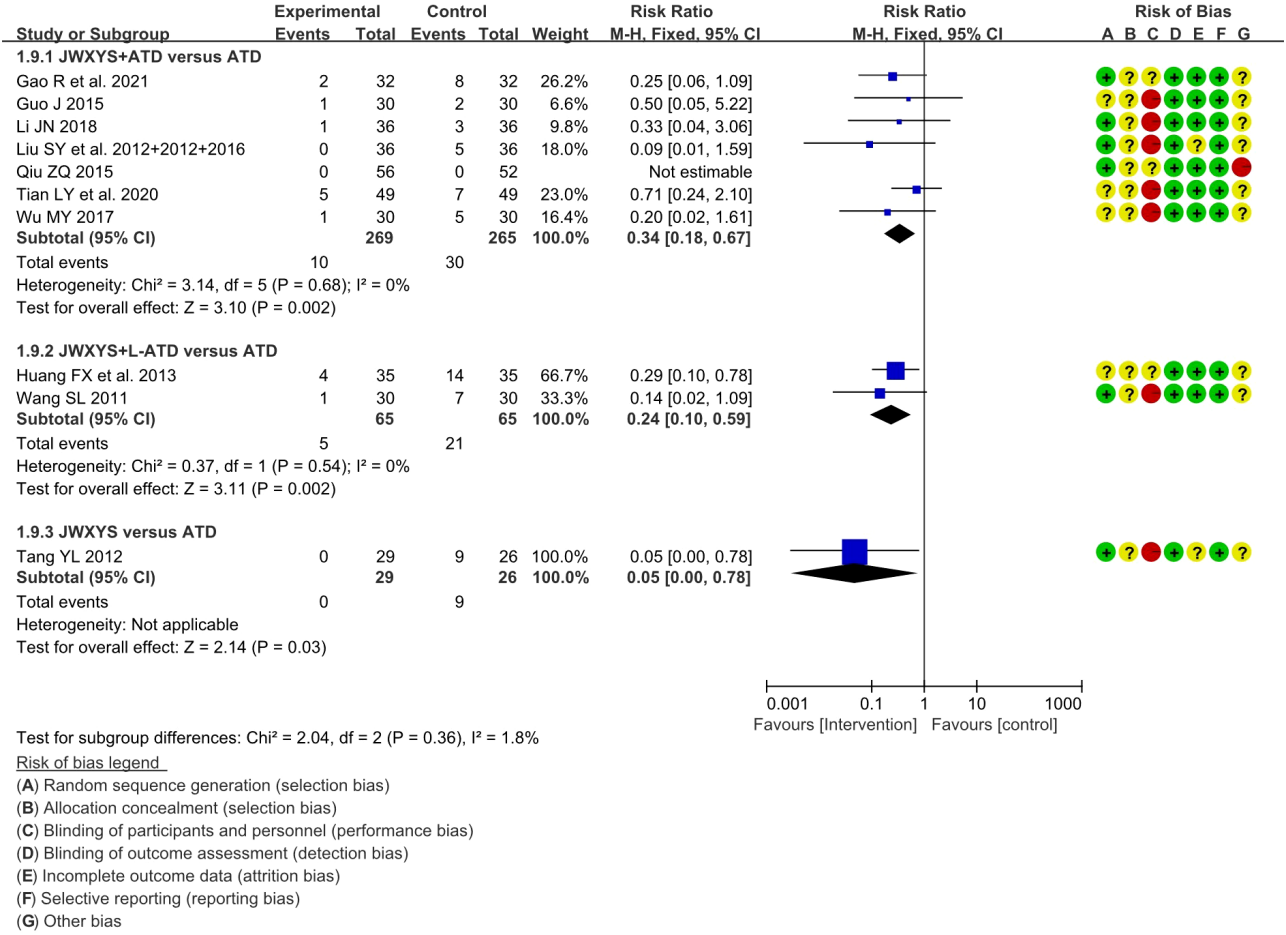
Forest plots of adverse events in different comparisons. JWXYS, Jiawei-Xiaoyao-San; ATD, antithyroid drug; L-ATD, half-dose antithyroid drug.

A total of 11 trials reported the AEs. Compared with ATDs, the combination of JWXYS and ATDs resulted in fewer AEs (RR = 0.34, 95% CI: 0.18 to 0.67; 7 trials, 534 participants), including less occurrence of hepatic dysfunction (14/303 versus 7/299), agranulocytosis (16/303 versus 9/299), and skin allergic reactions (6/303 versus 0/299). Compared with ATDs, the combination of JWXYS and half-dose ATDs resulted in fewer AEs (RR = 0.24, 95% CI: 0.10 to 0.59; 2 trials, 130 participants), including less occurrence of agranulocytosis (10/65 versus 3/65) and skin allergic reactions (11/65 versus 2/65). Compared with ATDs, mono-JWXYS resulted in fewer AEs (RR = 0.09, 95% CI: 0.01 to 1.58; 1 trial, 68 participants), including less occurrence of agranulocytosis (9/26 versus 0/29). The results of meta-analyses are summarized in **Table 2** and shown as forest plots in **Figure 7**.

#### 3.4.3 Sensitivity analyses

**Figure 8:**
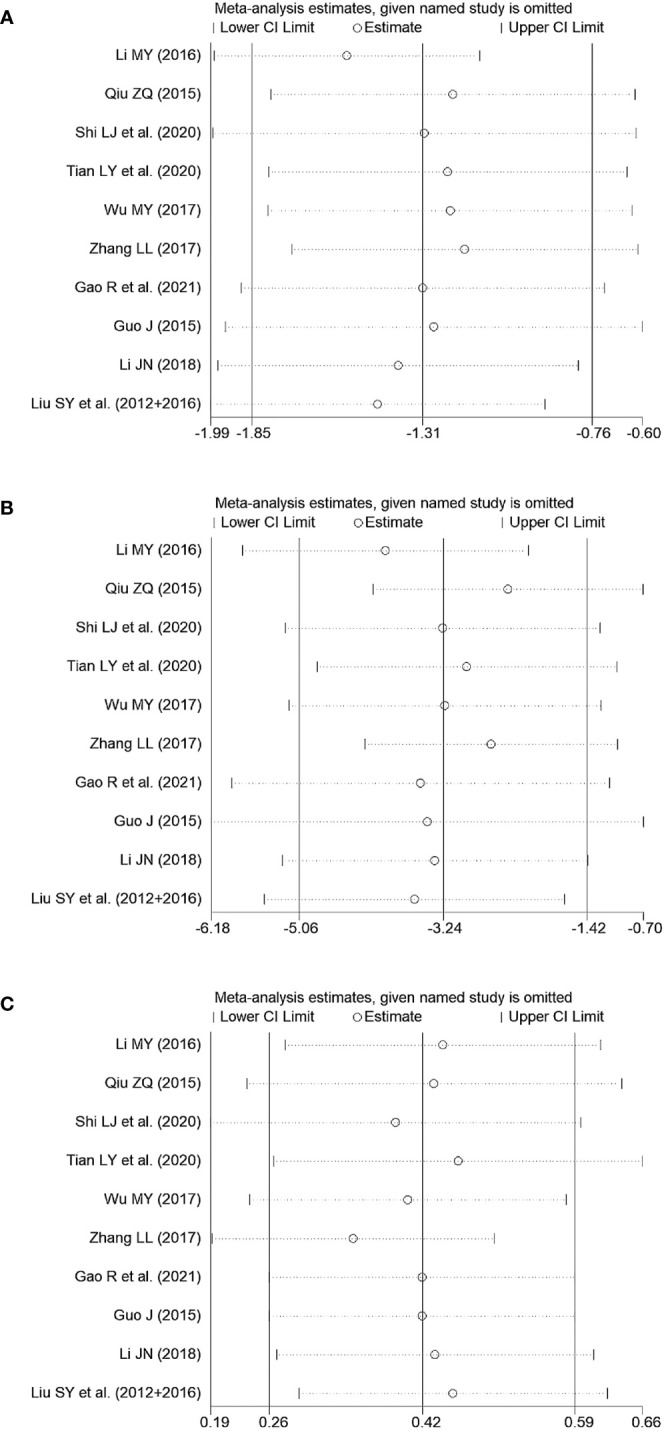
Sensitivity tests of primary outcomes in the comparison between Jiawei-Xiaoyao-San plus antithyroid drugs (ATDs) and mono-ATDs. **(A)**: Free triiodothyronine, **(B)**: Free thyroxine, **(C)**: Thyroid stimulating hormone.

Sensitivity analyses were conducted by removing each trial, which indicated that the pooled effects of primary outcomes in the comparison between JWXYS plus ATDs and mono-ATDs were robust (**Figure 8**).

#### 3.4.4 Subgroup analysis

**Figure 9:**
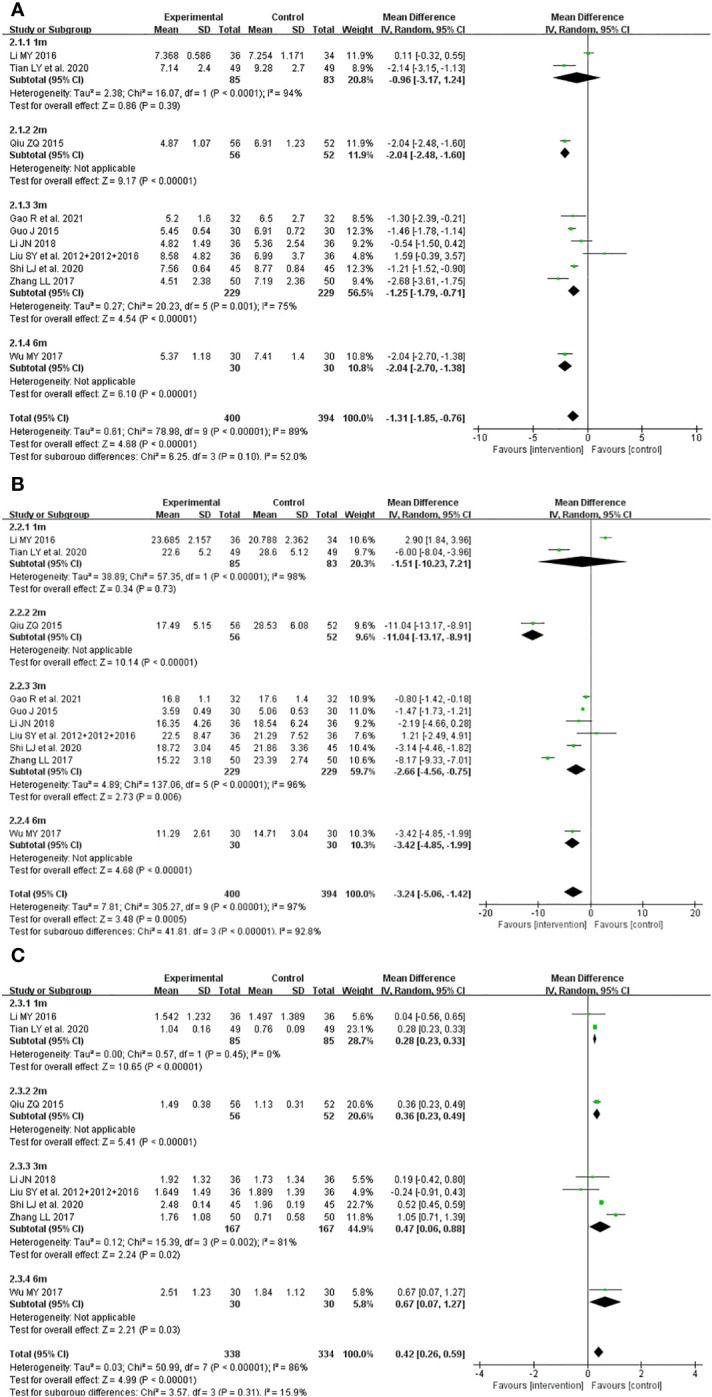
Subgroup analyses classified by course of intervention of primary outcomes in the comparison between Jiawei-Xiaoyao-San plus antithyroid drugs (ATDs) and mono-ATDs. **(A)**: Free triiodothyronine, **(B)**: Free thyroxine, **(C)**: Thyroid stimulating hormone.

The primary outcomes did not exhibit significant differences in subgroup analyses categorized by the course of intervention in the comparison between JWXYS plus ATDs and mono-ATDs (**Figure 9**), which may be attributed to the limited number of trials included in each subgroup.

### 3.5 Publication bias analysis

**Figure 10:**
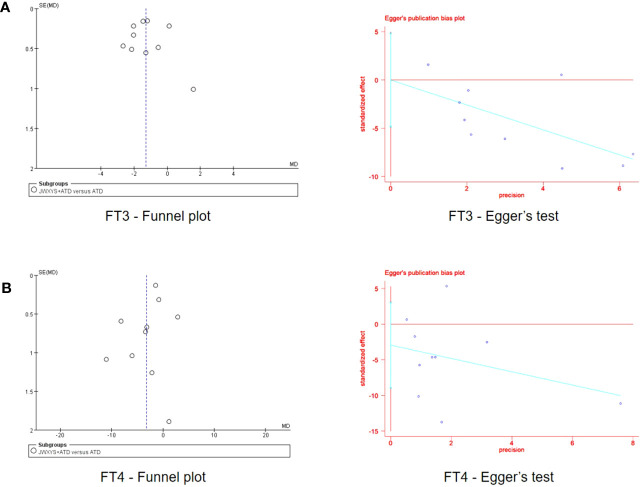
Funnel plots and Egger’s tests of the effectiveness on free triiodothyronine **(A)** and free thyroxine **(B)** reported by 10 included trials in the comparison between Jiawei-Xiaoyao-San plus antithyroid drugs (ATDs) and mono-ATDs.

Funnel plots were drawn to explore the possibility of publication bias for the ten trials comparing JWSYS plus ATDs with ATDs on the primary outcomes of FT3 and FT4. The scattered points distribution in the funnel plots was basically symmetrical. Egger’s tests were performed for further verification. Egger’s test of funnel plot asymmetry indicated almost no publication bias in the included studies (*P* = 0.997, *P* = 0.290), suggesting reliable results (**Figure 10**).

### 3.6 Certainty of evidence

Certainty of evidence and the reasons for the upgrade and downgrade are presented in **Table 2**. The certainty of evidence for each outcome was downgraded considering its risk of bias, inconsistency, indirectness, imprecision and other potential biases. The evidence for all outcomes were assessed as moderate to very low certainty. Moderate-certainty evidence showed that JWXYS plus ATDs resulted in fewer AEs. Low-certainty evidence suggests that JWXYS plus ATDs is more effective than ATDs in controlling thyroid functions (FT3, FT4, and TSH), alleviating TCM syndrome, and reducing goiter score and TRAb. The evidence of all other comparisons were assessed as low to very low certainty.

### 3.7 Quality of case reports

Three case reports containing three patients were included, and the quality of the reports was evaluated as high by the JBI tool (**Table 3**).

**Table 3:** Quality of case reports.

	Chang CC (2010)	Lin CH (2021)	Xia XG (2009)
1. Were patient’s demographic characteristics clearly described?	Yes	Yes	Yes
2. Was the patient’s history clearly described and presented as a timeline?	Yes	Yes	Yes
3. Was the current clinical condition of the patient on presentation clearly described?	Yes	Yes	Yes
4. Were diagnostic tests or assessment methods and the results clearly described?	Yes	Yes	Partially Yes
5. Was the intervention(s) or treatment procedure(s) clearly described?	Yes	Yes	Yes
6. Was the post-intervention clinical condition clearly described?	Yes	Yes	Yes
7. Were adverse events (harms) or unanticipated events identified and described?	Yes	Yes	No
8. Does the case report provide takeaway lessons?	Yes	Yes	Yes

### 3.8 Main findings of case reports

Reasons for patients seeking TCM-only treatment include suffering from or being afraid of severe AEs following ATDs and multiple relapses. Complete control of hyperthyroidism can be achieved with JWXYS therapy alone for 3 months to 3 years. The detailed components of the prescriptions used in each study are summarized in **Table 4**.

**Table 4:** Main findings of case reports.

Case	Author (Year)	Age and gender	Diagnosis and comorbidity	Symptoms	Laboratory tests	Treatment experience	Why choose TCM	TCM administration	Course of treatment	Outcomes	Long-term effect	Adverse events
1	Chang CC (2010)	33, female	Graves’ disease for 10years, Hepatitis B carrier	Palpitations, chest tightness, itching skin, string-like pulse and a thin pink tongue with thin white coating	Increased free T4, decreased TSH, increased TBII, and positive AMA	MMI (10mg, bid) for 4 months, MMI (5mg, bid to qd) for 1 year, PTU (100mg, bid)	Severe skin itching caused by ATDs and multiple relapses	Modified JWXYS as concentrated herbal extracts in powder form, orally, tid	3 years	The symptoms and thyroid functions alleviated after 3 months. T3 and T4 reached the normal range after 1 year. TSH returned to normal range another 1 year later.	No relapse in 3 years of follow-up after the discontinuance	Not occur
2	Lin CH (2021)	50, female	Graves’ disease for several weeks, Chocolate cysts, myomas, and chronic depression	Chest tightness, palpitations, hand tremors, excessive sweating and body weight loss	Abnormal thyroid function (free T4: 3.11; T3: 365.39; TSH <0.01; anti-TPO: 1748.38; anti-THYG: 288.90; anti-TSHR: 3.2	Accepted both ATD and TCM therapy, initially	Afraid of side effects caused by ATDs	Modified JWXYS as concentrated herbal extracts in powder form, orally, tid	1 year	The symptoms alleviated after 2 months. After 10 months, thyroid hormone levels were within the normal range.	No relapse in 1 year of follow-up	Not occur
3	Xia XG (2009)	39, female	Hyperthyroidism for 6 months	Irritability, palpitation, insomnia, dreaminess, chest tightness, belching, fatigue, dizziness, excessive sweating, trembling hands, low menstrual volume, bitter mouth, red tongue with thin and yellow coating, stringy pulse	T3, T4 > normal range; WBC: 2.5×10^9^/L; Bp: 145/92mmHg; HR: 104/min; mild goiter	Treated with methimazole, vitamin B4 and hemogenesis.	Leukocytes decreased after treatment with MMI, but did not recover after treatment with leucogen tablets. The patient feared this side effect.	Modified JWXYS decoction, Jiawei Xiaoyao Pills, orally, tid	40 days of decoction, and then a month of Jiawei Xiaoyao Pills	After 15 days, most symptoms were significantly reduced. After 40 days, all symptoms disappeared, and the thyroid function and leukocytes were in the normal range. Bp: 128/80mmHg; HR: 84/min; No goiter	No relapse in 6 years of follow-up after the discontinuance	Not reported

T3, triiodothyronine; T4, tetraiodothyronine; TSH, thyroid stimulating hormone; MMI, Methimazole; PTU, propylthiouracil; anti-TPO, thyroid peroxidase antibodies; anti-THYG, anti-thyroglobulin antibodies; AMA, antithyroid microsomal antibodies; WBC, white blood cell count; TBII, thyrotropin binding inhibiting immunoglobulin; Bp, blood pressure; HR, Heart rate; tid, 3 times a day; bid, 2 times a day; qd, 1 time a day.

### 3.9 Summary of pharmacological studies

Three experiments in seven reports were included (45–51), and the characteristics and main findings were summarized in **Table 5**. Wu XY’s study (49) showed that JWXYS plus *Anemone flaccida Fr. Schmidt* (Diwu) regulated T cell differentiation and restored Th17/Treg cell balance by inhibiting the JAK1-STAT3 pathway of CD4^+^ T cells. Bao CY et al. (45) found that modified JWXYS plus PTU reduced the incidence of adverse pregnancy in female mice, as well as lowered thyroid function and activity of deiodinases of offspring mice. Tan HZ et al. (46–48, 50, 51) found that modified JWXYS could regulate the proliferation and apoptosis of thyroid cells and protect liver function by alleviating oxidative stress. The detailed components of the prescriptions used in each study are summarized in the **Supplementary Material**.

**Table 5:** Characteristics and main findings of mechanism studies.

Author (Year)	Animal	Modeling inducer	Intervention	Effects for disease	Pharmacological findings
Wu XY (2021)	SD female Rat	Rat thyroid membrane protein, i.h, three times	JWXYS + Anemone flaccida Fr. Schmidt (Diwu)	T3↓, T4↓, TRAb↓, Thyroid cell hyperplasia↓	CD4^+^/CD8^+^ on thyroid cells↓; IFN-γ, IL-10, IL-17 and IL-21 in serum↓; JAK1, STAT3, p-STAT3, RORγt, FOXP3 and RORyt/FOXP3 in spleen↓
Bao CY et al. (2019)	C57/BL6 female mice and their offspring	Ad-TSHR289/6xHis adenovirus, three times	Modified JWXYS + PTU	Incidence of adverse pregnancy in female mice↓; Body mass of offspring mice↑; FT3, FT4, TT3 and TT4 of offspring mice↓	Activity of deiodinases (type I, II, III) in the offspring mice↓
Tan HZ et al. (2017)	BALB/c female mice	pAdCMV-TSHR289/Flag (Dsredexpress2) recombinant adenovirus, i.m, three times	Modified JWXYS	T3↓, T4↓, TRAb↓, TSH↑; Food intake↓, water intake↓, body temperature↓; Thyroid cell hyperplasia↓; Pathological lesion of liver tissue	Bcl-2 in thyroid↑; Caspase-3, BAX in thyroid↓; AST, ALT, ALP in serum↓; MDA, SOD, CAT, GSH-PX, Nrf-2, HO-1, 8-OHDG in liver↓

IFN-γ, interferon gamma; IL, interleukin; JAK1, Janus kinase one; FOXP3, Forkhead box protein P3; STAT3, signal transducer and activator of transcription three; RORγt, retinoic acid related orphan receptor gamma (t); Bcl-2, B-cell lymphoma-2; BAX, BCL2-Associated X; AST, aspartate aminotransferase; ALT, alanine transaminase; ALP, alkaline phosphatase; MDA, malondialdehyde; SOD, superoxide dismutase; CAT, catalase; GSH-PX, glutathione peroxidase; Nrf-2, nuclear factor erythroid 2-related factor 2; HO-1, heme oxygenase-1; 8-OHDG, 8-hydroxy-2 deoxyguanosin.

## 4 Discussion

### 4.1 Summary of evidence

Thirteen RCTs involving 979 participants on JWXYS for hyperthyroidism were included. We found that compared with ATDs, the complementary treatment of JWXYS could help control thyroid functions, alleviate TCM syndrome and goiter, and reduce TRAb and AEs. Meanwhile, when combined with JWXYS, half-dose of ATDs could ideally control thyroid function, thus significantly reducing the occurrence of AEs, and were also effective in alleviating TCM syndrome and goiter. But the effectiveness of mono-JWXYS in controlling thyroid function was significantly inferior to that of ATDs. In cases of severe adverse reactions following ATDs, alternative therapy with JWXYS may be attempted, which may allow complete control of hyperthyroidism. Sensitivity analyses were conducted by removing each trial, which indicated that the pooled effects of primary outcomes were robust. The risk of bias was high among the studies, mainly due to the lack of description of the randomization process and the absence of blinding. Funnel plots and Egger’s tests indicated no existence of publication bias. The majority of the evidence were assessed as moderate to very low certainty.

### 4.2 Administration of JWXYS for hyperthyroidism

JWXYS can be used to treat a variety of thyroid disorders, such as Hashimoto’s thyroiditis (52), subacute thyroiditis (53), thyroid-related eye diseases (54), nodular goiter (55), etc., especially for the treatment of hyperthyroidism. In TCM theory, patients with hyperthyroidism are usually diagnosed as the syndrome of liver depression inducing fire, the syndrome of hyperactivity of fire due to yin deficiency or the syndrome of hyperactivity of heart-liver fire. As a classical CHM prescription, JWXYS is commonly used in the treatment of the syndrome of liver depression inducing fire and spleen deficiency. It is often used to relieve various symptoms, such as anxiety, depression, hot flashes, night sweating, palpitations, headache, dry eyes, sleep disturbance, rash, and irregular menstruation, all of which are just in line with the characteristics of hyperthyroidism.

JWXYS formula contains ten herbs: *Paeonia suffruticosa Andr.* (Danpi), *Gardenia jasminoides Ellis* (Zhizi)*, Angelica sinensis (Oliv.) Diels* (Danggui), *Paeonia lactiflora Pall.* (Baishao), *Bupleurum chinense DC.* (Chaihu), *Poria cocos (Schw.) Wolf* (Fuling), *Atractylodes macrocephala Koidz.* (Baizhu), *Glycyrrhiza uralensis Fisch* (Gancao), *Mentha haplocalyx Briq.* (Bohe), *Zingiber officinale Rosc.* (Shengjiang). In clinical practice, JWXYS is often modified to target different symptoms of patients. In the included studies, *Prunella vulgaris* (Xiakucao), *Scutellariae Radix* (Huangqin), *Lycii Cortex* (Digupi), *Ziziphi Spinosae Semen* (Suanzaoren), and *Figwort Root* (Xuanshen) are the most commonly added herbs, while *Zingiber Officinale Roscoe* (Shengjiang) and *Menthae Herba* (Bohe) are often removed. In case of significant goiter, *Fritillariae Thunbrgii Bulbus* (Beimu), Xuanshen, *Ostrea gigas Thunberg* (Muli), and particularly Xiakucao, are often added (16, 23, 37, 43). Xiao-Luo-Wan, which consists of Beimu, Xuanshen and Muli, is also one of the classic TCM prescriptions and is mainly used to treat a variety of proliferative diseases, including goiter and thyroid nodule. Xiakucao is one of the most commonly used Chinese herbal medicines in the treatment of GD, which can be used either alone or in a Chinese herbal formula. Many clinical trials have verified their effectiveness, and several pharmacological studies have been conducted (56, 57). When the symptoms of fear of heat, dry mouth and hyperhidrosis are obvious, huangqin or Digupi is often added to treat TCM syndrome of fire of excessive type and deficiency type, respectively. Suanzaoren is often added to treat insomnia.

In addition to the studies and outcomes included in this systematic review, the existing literature also reported other applications and effects of JWXYS in the treatment of hyperthyroidism. Liu XX et al. (58) reported that after microwave ablation, the application of JWXYS can significantly relieve clinical symptoms such as palpitations and irritability, and help control thyroid functions. Liu HJ et al. (59) have shown that the application of JWXYS to patients with hyperthyroidism during pregnancy can improve pregnancy outcomes, such as reducing postpartum blood loss, natural labor rate, and postpartum infection rate. Furthermore, studies have shown that JWXYS can reduce the Hamilton Depression Scale score (31) and bilateral superior thyroid artery systolic peak flow velocity (60) in patients with hyperthyroidism. The above studies were not included in this systematic review for meta-analysis because they did not meet the eligibility criteria and the frequency of outcome reporting was low.

### 4.3 Pharmacological mechanism of JWXYS for hyperthyroidism

Hyperthyroidism is mainly caused by GD, and the core of its pathogenesis is the loss of self-tolerance to TSHR, resulting in the production of stimulating thyrotropin receptor antibodies. Three experiments explored the mechanism of JWXYS in the treatment of hyperthyroidism. All three studies were conducted in animals to simulate the onset of GD by injecting antigen (thyroid membrane protein, adenovirus or recombinant adenovirus packaging TSHR plasmid), and all used a three-dose procedure. The key to the mechanism study is the success of the disease model, and the modeling rate and the maintenance of the disease state seriously affect the results of the intervention experiment. All the studies evaluated the success of the modeling, however, none of the three studies reported the modeling rate. The recombinant adenovirus packaged with the TSHR289 plasmid in Tan HZ et al.’s study (46–48, 50, 51) was from our team. In recent years, our team has further optimized the modeling method. The improved single injection method has shortened the modeling time, and the modeling rate can reach 100% within 4 weeks after injection (61).

When GD occurs, the inhibitory function and/or proportion of regulatory T cells (Treg) is decreased, and helper T cells (Th) are abnormally activated. The increased ratio of Th1 and Th17 secretes cytokines such as interferon gama (IFN-γ) and interleukin (IL) 17, thus promoting recruitment and differentiation of B cells and plasma cells. Wu XY’s study (49) shows that JWXYS derived formula can regulate T cell differentiation and restore Th17/Treg cell balance in the treatment of GD by inhibiting JAK1-STAT3 pathway of CD4^+^T cells.

Abnormal thyroid function is an important cause of adverse pregnancy outcomes and has adverse effects on the health of offspring (62). Bao CY’s study shows that modified JWXYS reduced the incidence of adverse pregnancy in female mice, and meanwhile JWXYS reduced the secretion of thyroid hormones and activity of deiodinases (type I, II, III) in the offspring mice.

Under normal conditions, the proliferation and apoptosis of thyroid cells are balanced to maintain the morphological stability. Under the stimulation of TRAb, the proliferation and apoptosis of thyroid cells were unbalanced, resulting in hyperplasia and proliferation. Tan HZ et al.’s studies (46) showed that modified JWXYS regulated the imbalance of a pair of markers of proliferation and apoptosis (Bcl-2 and BAX). Oxidative stress is a characteristic of hyperthyroidism and can lead to hepatic damage. JWXYS reduced the concentration of aspartate aminotransferase (AST), alanine transaminase (ALT) and alkaline phosphatase (ALP) in serum (47, 48) and 8-hydroxy-2 deoxyguanosine (8-OHDG) expression in the liver (51). Meanwhile, modified JWXYS reduced the levels of malondialdehyde (MDA), superoxide dismutase (SOD) and catalase (CAT) in the liver of GD mice (46–48, 51), indicating the restoration of the balance of oxidation/antioxidant system, which is superior to methimazole. Modified JWXYS could regulate the expression of mRNA and protein of nuclear factor erythroid 2-related factor 2(Nrf2) and heme oxygenase-1(HO-1), the key factors of oxidative stress pathway (46). Therefore, modified JWXYS could protect liver function by alleviating oxidative stress in GD mice.

A network pharmacological study (49) predicted that the main active ingredients of JWXYS in GD treatment include (+)-catechin, beta-sitosterol, formononetin, kaempferol, licochalcone A, naringenin, paeoniflorin, quercetin, shinpterocarpin, sitosterol, stigmasterol and sudan III. JWXYS may intervene in inflammation, immunity, apoptosis and other mechanisms to treat Graves’ disease through IL-17 signaling pathway, TNF signaling pathway, cytokine - cytokine receptor interaction and other signaling pathways. Validation is still needed *in vivo* and *in vitro*.

### 4.4 Strengths and limitations

To our knowledge, this is the first systematic review to evaluate the effectiveness and safety of JWXYS in the treatment of hyperthyroidism. A comprehensive search of eight databases and three trial registries was conducted to ensure that all RCTs were included, and the methodology was reported in detail and transparently to allow for replication. At the same time, we also searched for the case reports of JWXYS alone for the treatment of hyperthyroidism, and summarized the reasons for patients seeking this treatment and its effectiveness, providing a possible alternative therapy for the treatment of hyperthyroidism. In addition, we also searched and summarized the pharmacological mechanism of JWXYS in the treatment of hyperthyroidism. This systematic review provides an effective and safe TCM complementary and alternative therapy for the treatment of hyperthyroidism.

However, there are several limitations in this systematic review. The included studies were all published in Chinese and only Chinese participants were recruited, so the generalizability of the findings was limited. All of the included studies were of low quality, with small sample sizes and without rigorous experimental protocols and follow-up. None of the studies reported relapse rates. Due to the insufficient number of trials included, some comparisons could not be pooled for meta-analysis, and subgroup analyses classified by course of intervention did not show significant differences in primary outcomes. But some trials showed that the adjuvant of JWXYS achieved quicker effectiveness, especially in alleviating symptoms (41). In addition, all studies administered modified JWXYS with different drugs and dosages, and the symptoms and signs included in various studies were not exactly the same, resulting in clinical heterogeneity. Publication bias assessment was performed only for primary outcomes in the JWXYS plus ATDs and ATDs comparisons, as few studies could be included in other outcomes and comparisons.

### 4.5 Implications

Future studies with appropriate randomization and double-blind methods are warranted to confirm these findings. The CONSORT checklist (63) is recommended for researchers when drafting RCTs. In the literature screening, it was found that some studies only took clinical effective rate as the primary outcome, and did not report thyroid function data. Single objective outcome is recommended as the primary outcome, rather than the compound outcome of clinical effectiveness. Thyroid volume data obtained from thyroid color ultrasound should be used to explore the improvement of goiter, rather than visual judgment of appearance. It is necessary to establish a core outcome set and promote its application to standardize the trial design, so as to reduce the clinical heterogeneity of meta-analysis. More studies are needed to explore the effect of JWXYS on TRAb, and follow-up should be conducted after treatment to obtain recurrence rates. When conducting a pharmacological mechanism study, it is essential to analyze the components of the drugs utilized. A more robust modeling approach should be employed and the success rate should be confirmed in future animal experiments to ensure the stability and reliability of the results.

## 5 Conclusions

The current evidence suggests that JWXYS may enhance the effectiveness of ATDs in managing hyperthyroidism, particularly with regards to symptom relief and goiter reduction. Meanwhile, JWXYS can reduce AEs caused ATDs. Mono-JWXYS is not recommended as a therapeutic alternative, except in patients who cannot tolerate ATDs. However, the findings should be interpreted with caution due to overall high risk of bias. Future studies with appropriate randomization and double-blind methods are warranted to confirm these findings. Further pharmacological studies with more reliable models are needed *via in vivo* and *in vitro* studies.

## Data availability statement

The original contributions presented in the study are included in the article/**Supplementary Material**. Further inquiries can be directed to the corresponding author.

## Author contributions

W-XM and YT conceived and designed the review. W-XM drafted the manuscript. GW and M-YX conducted the data collection. R-TZ and W-XM participated in data extraction and study risk of bias assessment. P-YP and W-XM performed the statistical analysis. L-KL prepared the figures and completed the PRISMA checklist. Z-LL, J-PL, X-WZ and X-YZ were involved in critically revising the manuscript. All authors contributed to the article and approved the submitted version.

## References

[1] McDermott MT . Hyperthyroidism. Ann Internal Med (2020) 172(7):Itc49–itc64. doi: 10.7326/aitc202004070 32252086

[2] Ross DS Burch HB Cooper DS Greenlee MC Laurberg P Maia AL . 2016 American thyroid association guidelines for diagnosis and management of hyperthyroidism and other causes of thyrotoxicosis. Thyroid: Off J Am Thyroid Assoc (2016) 26(10):1343–421. doi: 10.1089/thy.2016.0229 27521067

[3] De Leo S Lee SY Braverman LE . Hyperthyroidism. Lancet (London England) (2016) 388(10047):906–18. doi: 10.1016/s0140-6736(16)00278-6 PMC5014602 27038492

[4] Chen Q Yan Y Zhang L Cheng K Liu Y Zhu W . Effect of hyperthyroidism on the hypercoagulable state and thromboembolic events in patients with atrial fibrillation. Cardiology (2014) 127(3):176–82. doi: 10.1159/000356954 24434544

[5] Selmer C Olesen JB Hansen ML von Kappelgaard LM Madsen JC Hansen PR . Subclinical and overt thyroid dysfunction and risk of all-cause mortality and cardiovascular events: A large population study. J Clin Endocrinol Metab (2014) 99(7):2372–82. doi: 10.1210/jc.2013-4184 24654753

[6] McLeod DS Caturegli P Cooper DS Matos PG Hutfless S . Variation in rates of autoimmune thyroid disease by race/ethnicity in us military personnel. Jama (2014) 311(15):1563–5. doi: 10.1001/jama.2013.285606 24737370

[7] Wiersinga WM . Graves' Disease: can it be cured? Endocrinol Metab (Seoul Korea) (2019) 34(1):29–38. doi: 10.3803/EnM.2019.34.1.29 PMC6435849 30912336

[8] Davies TF Andersen S Latif R Nagayama Y Barbesino G Brito M . Graves' Disease. Nat Rev Dis Primers (2020) 6(1):52. doi: 10.1038/s41572-020-0184-y 32616746

[9] Cramon P Winther KH Watt T Bonnema SJ Bjorner JB Ekholm O . Quality-of-life impairments persist six months after treatment of graves' Hyperthyroidism and toxic nodular goiter: A prospective cohort study. Thyroid: Off J Am Thyroid Assoc (2016) 26(8):1010–8. doi: 10.1089/thy.2016.0044 27370744

[10] Mirallié E Borel F Tresallet C Hamy A Mathonnet M Lifante JC . Impact of total thyroidectomy on quality of life at 6 months: the prospective thyrqol multicentre trial. Eur J Endocrinol (2020) 182(2):195–205. doi: 10.1530/eje-19-0587 31804967

[11] Törring O Watt T Sjölin G Byström K Abraham-Nordling M Calissendorff J . Impaired quality of life after radioiodine therapy compared to antithyroid drugs or surgical treatment for graves' Hyperthyroidism: A long-term follow-up with the thyroid-related patient-reported outcome questionnaire and 36-item short form health status survey. Thyroid: Off J Am Thyroid Assoc (2019) 29(3):322–31. doi: 10.1089/thy.2018.0315 30667296

[12] Brito JP Payne S Singh Ospina N Rodriguez-Gutierrez R Maraka S Sangaralingham LR . Patterns of use, efficacy, and safety of treatment options for patients with graves' Disease: A nationwide population-based study. Thyroid: Off J Am Thyroid Assoc (2020) 30(3):357–64. doi: 10.1089/thy.2019.0132 31973681

[13] Ma C Xie J Wang H Li J Chen S . Radioiodine therapy versus antithyroid medications for graves' Disease. Cochrane Database systematic Rev (2016) 2:Cd010094. doi: 10.1002/14651858.CD010094.pub2 PMC10517434 26891370

[14] Azizi F Abdi H Mehran L Amouzegar A . Appropriate duration of antithyroid drug treatment as a predictor for relapse of graves' Disease: A systematic scoping review. J Endocrinol Invest (2022) 45(6):1139–50. doi: 10.1007/s40618-021-01730-1 35088381

[15] Burch HB Cooper DS . Anniversary review: antithyroid drug therapy: 70 years later. Eur J Endocrinol (2018) 179(5):R261–r74. doi: 10.1530/eje-18-0678 30320502

[16] Lin CH Lin CP Huang ST . Successful intervention with chinese herbal medicine for hyperthyroidism: two case reports and a literature review. Explore (New York NY) (2021) 17(4):344–50. doi: 10.1016/j.explore.2020.10.007 33109498

[17] Kim J Kim TH . A methimazole resistant patient with graves' Disease (Gd): A case report of mid-term management with herbal decoctions mainly composed of anemarrhena bunge. Complement Ther Med (2018) 39:109–13. doi: 10.1016/j.ctim.2018.05.015 30012381

[18] Taïbi K Ait Abderrahim L Helal F Hadji K . Ethnopharmacological study of herbal remedies used for the management of thyroid disorders in Algeria. Saudi Pharm journal: SPJ Off Publ Saudi Pharm Soc (2021) 29(1):43–52. doi: 10.1016/j.jsps.2020.12.004 PMC7873729 33603538

[19] He Q Dong H Gong M Guo Y Xia Q Gong J . New therapeutic horizon of graves' Hyperthyroidism: treatment regimens based on immunology and ingredients from traditional chinese medicine. Front Pharmacol (2022) 13:862831. doi: 10.3389/fphar.2022.862831 35462920 PMC9020194

[20] Chang C-C Wu S-Y Lai Y-R Hung Y-C Hsu CY Chen H-J . The utilization of chinese herbal products for hyperthyroidism in national health insurance system (Nhird) of Taiwan: A population-based study. Evidence-Based Complementary Altern Med (2022) 2022:5500604. doi: 10.1155/2022/5500604 PMC9017513 35449810

[21] Huang FX Chen CG Wu MF . Clinical study of danzhi xiaoyao pill in treating hyperthyroidism induced liver injury [Article in chinese]. Hebei Traditional Chin Med (2013) 35(02):187–9. doi: 10.3969/j.issn.1002-2619.2013.02.011

[22] Gao R Hu JZ . Clinical observation of danzhi xiaoyao powder in the treatment of irate exuberant syndrome of graves disease. Clin J Traditional Chin Med (2021) 33(04):730–3. doi: 10.16448/j.cjtcm.2021.0434

[23] Chang CC Huang ST . Is traditional chinese medicine effective for reducing hyperthyroidism? J Altern complementary Med (New York NY) (2010) 16(11):1217–20. doi: 10.1089/acm.2010.0114 21058888

[24] Xia XG . Using danzhi xiaoyao powder to treat hyperthyroidism. Sichuan Traditional Chin Med (2009) 27(06):95.

[25] Higgins JPT TJ Chandler J Cumpston M Li T Page MJ Welch VA . Cochrane handbook for systematic reviews of interventions version 6.3. Cochrane (2022). 10.1002/14651858.ED000142 PMC10284251 31643080

[26] Page MJ McKenzie JE Bossuyt PM Boutron I Hoffmann TC Mulrow CD . The prisma 2020 statement: an updated guideline for reporting systematic reviews. BMJ (2021) 372:n71. doi: 10.1136/bmj.n71 33782057 PMC8005924

[27] Zheng X . Guiding principle of clinical research on new drugs of traditional chinese medicine . China Medical Science and Technology Press. (2002) 229.

[28] Institute TJB . Joanna briggs institute reviewers’ Manual. In: The joanna briggs institute. The joanna briggs institute, 2016 Edition (2016), 3. Available at: https://jbi.global/critical-appraisal-tools.

[29] Shi J Luo D Weng H Zeng XT Lin L Chu H . Optimally estimating the sample standard deviation from the five-number summary. Res synthesis Methods (2020) 11(5):641–54. doi: 10.1002/jrsm.1429 32562361

[30] Egger M Davey Smith G Schneider M Minder C . Bias in meta-analysis detected by a simple, graphical test. BMJ (Clinical Res ed) (1997) 315(7109):629–34. doi: 10.1136/bmj.315.7109.629 PMC2127453 9310563

[31] Liu SY . Clinical observation on the treatment of blood glucose, blood lipid and depression in patients with hyperthyroidism (Type of pathogenic fire derived from stagnation of liver-qi) by soothing the liver and puring fire. [Master Dissertation]. Chengdu, China: Chengdu University of Traditional Chinese Medicine (2012).

[32] Zhang LL . Liver hydrophobic heat method integrated clinical intervention for patients with hyperthyroidism. [Master Dissertation]. Chengdu, China: Chengdu University of Traditional Chinese Medicine (2017).

[33] Fu XX Li HY Kang XY Liu SY Zhang JJ Xie CG . Clinical study on danzhi compound in treating hyperthyroidism (Type of liver depression inducing fire). J Sichuan Traditional Chin Med (2016) 34(03):70–3.

[34] Tian LY Zhang GL He H Cao J Wang LY . Clinical trial of danzhixiaoyao powder combined with thiamazole in treating hyperthyroidism [Article in chinese]. Hunan J Traditional Chin Med (2020) 36(11):62–4. doi: 10.16808/j.cnki.issn1003-7705.2020.11.023

[35] Shi LJ Liu J Zhang XR Ren XY Zhao LL Li Q . Clinical study of danzhi xiaoyao powder combined with methimazole in treating hyperthyroidism. Shanxi J Traditional Chin Med (2020) 41(09):1276–8+300. doi: 10.3760/cma.j.issn.1674-4756.2015.06.058

[36] Li JN . Clinical study on the treatment of hyperthyroidism (Liver-fire exuberance syndrome) by inhibiting yang and strengthening yin. [Master Dissertation]. Chengdu, China: Chengdu University of Traditional Chinese Medicine (2018).

[37] Wu MY . Clinical observation on effect of danzhixiaoyao powder in the treatment of hyperthyroidism. [Master Dissertation]. Guangzhou, China: Guangzhou University of Traditional Chinese Medicine (2017).

[38] Li MY . Effects of danzhi compound on the thyroid function in mice with graves’ Hyperthyroidism and patients with hyperthyroidism characterized with pathogentic fire derived from stagnation of liver-qi. [Master Dissertation]. Chengdu university of traditional chinese medicine (2016).

[39] Li L . Clinical curative effect observation of danzhixiaoyaosan on treating hyperthyroidism (the type of heart-fire and liver-fire). [Master Dissertation]. Chengdu, China: Chengdu University of Traditional Chinese Medicine (2016).

[40] Qiu ZQ Qian HQ . Clinical observation of xiaoyao powder combined with western medicine in treating hyperthyroid cardiopathy. J Emergency Traditional Chin Med (2015) 24(05):916–7.

[41] Guo J . Clinical observation of the treatment of graves' Disease by using dan zhi xiaoyao powder. [Master Dissertation]. Hubei University of Traditional Chinese Medicine (2015).

[42] Zhang JJ . Clinical observation on the method of soothing liver and clearing heat in the treatment of patients with hyperthyroidism (Type of liver depression inducing fire). [Master Dissertation]. Chengdu, China: Chengdu University of Traditional Chinese Medicine (2012).

[43] Wang SL . Clinical research of danzhi xiaoyao powder modified thismazole on hyperthyroidism. [Master Dissertation]. Guangzhou, China: Jinan University (2011).

[44] Tang YL . Clinical observation of the effect of dan zhi xiao yao decoction on the patients with liver stagnation and spleen deficiency and heat of hashimoto's thyroiditis and hyperthyroidism. [Master Dissertation]. Jinan, China: Shandong University of Traditional Chinese Medicine (2012).

[45] Bao CY Zhou LY Yu XX Li CX . Effects of propyl thiouracil combined with danzhi compound on thyroid function in the progeny of mice with hyperthyroidism and pregnancy. J Guangzhou Univ Chin Med (2019) 36(07):1059–63. doi: 10.13359/j.cnki.gzxbtcm.2019.07.026

[46] Tan HZ . Study on dose-effect and time-effect relationship and mechanism of danzhi compound in treating graves disease mice in the view of "Liver". [Doctoral Dissertation]. Chengdu university of traditional chinese medicine (2017).

[47] Liu J . Effect of danzhi compound on oxidative stress response of liver injury in hyperthyroidism model mice. [Master Dissertation]. Chengdu university of traditional chinese medicine (2017).

[48] Liu Q . Effects of danzhi compound on thyroid function, liver injury and oxidative stress in gd mice. [Master Dissertation]. Chengdu university of traditional chinese medicine (2017).

[49] Wu XY . Preliminary study on the efficacy and pharmacology of jiawei xiaoyao pills in treating graves' Disease. [Master Dissertation]. Peking Union Medical College (2021).

[50] Huang YW . Effect of danzhi compound on the expression of thyroid caspase-3, bcl-2 and bax protein in mice with graves' Disease. [Master Dissertation]. Chengdu university of traditional chinese medicine (2017).

[51] Jia YL . Effects of danzhi compound on thyroid function and DNA oxidative damage of hepatocytes in hyperthyroidism model mice. [Master Dissertation]. Chengdu university of traditional chinese medicine (2017).

[52] Du JH Bing LL Xie YR Yu WJ . Clinical observation on hypothyroidism of hashimoto's thyroiditis treated with xiakucao granules and danzhi xiaoyao san granules together. Beijing J Traditional Chin Med (2020) 39(07):738–41. doi: 10.16025/j.1674-1307.2020.07.022

[53] Cui Y . Observation on the curative effect of jiawei xiaoyao san combined with indomethacin in the treatment of subacute thyroiditis. Shaanxi J Traditional Chin Med (2015) 36(09):1177–8. doi: 10.3969/j.issn.1000-7369.2015.09.038

[54] Fan YF Yue L Deng AM Jie LG Song DD . Effect of danzhi-xiaoyao san on tear th1/th2 cytokines levels in patients with active thyroid-associated ophthalmopathy. Chin Pract Med (2020) 15(11):1–4. doi: 10.14163/j.cnki.11-5547/r.2020.11.001

[55] Gao YH Wang DC Zhao HX . Clinical effect of modified dan zhi xiaoyao powder combined with xiaojin pills for treatment of nodular goiter. J Guangzhou Univ Traditional Chin Med (2016) 33(06):810–2. doi: 10.13359/j.cnki.gzxbtcm.2016.06.013

[56] Dong S Liu Q Jiang M Ma Q Huang Q Liu T . Xiao-luo-wan treats propylthiouracil-induced goiter with hypothyroidism in rats through the pi3k-akt/ras pathways based on uplc/ms and network pharmacology. J Ethnopharmacol (2022) 289:115045. doi: 10.1016/j.jep.2022.115045 35101570

[57] Zhang W Wuhan Q Na M Hu R Mu Q Bao X . Emerging therapeutic role of prunella vulgaris in thyroid disease. Chin Herbal Med (2022) 14(3):403–13. doi: 10.1016/j.chmed.2021.12.005 PMC9476742 36118009

[58] Liu XX Ye JH Wang JL He DY . Effect of danzhi xiaoyao powder in treatment of graves disease in women of childbearing age. Chin Arch Traditional Chin Med (2022) 40(05):216–9. doi: 10.13193/j.issn.1673-7717.2022.05.050

[59] Liu HJ LXL Liu QJ Bian RR . Clinical study on modified danzhi xiaoyao powder combined with propylthiouracil for gestational hyperthyroidism of intense liver fire type. New Chin Med (2021) 53(22):33–6. doi: 10.13457/j.cnki.jncm.2021.22.009

[60] Pan JX He XL Zhang J Wan R Cai LS Li Y,Y . Observation of curative effect of danzhi xiaoyao powder combined with western medicine in the treatment of hyperthyroidism and its influence on hemodynamics. New Chin Med (2016) 48(12):52–4. doi: 10.13457/j.cnki.jncm.2016.12.023

[61] Tang Y Zhu X Feng H Zhu L Fu S Kong B . An improved mouse model of graves disease by once immunization with ad-tshr289. Endocr J (2019) 66(9):827–35. doi: 10.1507/endocrj.EJ19-0148 31217394

[62] Andersen SL Knøsgaard L . Management of thyrotoxicosis during pregnancy. Best Pract Res Clin Endocrinol Metab (2020) 34(4):101414. doi: 10.1016/j.beem.2020.101414 32199749

[63] Schulz KF Altman DG Moher D . Consort 2010 statement: updated guidelines for reporting parallel group randomised trials. BMJ (2010) 340:c332. doi: 10.1136/bmj.c332 20332509 PMC2844940

