# Supplementary material for: Effectiveness and potential mechanism of Jiawei-Xiaoyao-San for hyperthyroidism: a systematic review
Source: Front Endocrinol (Lausanne). 2023 Sep 13;14:1241962. doi: 10.3389/fendo.2023.1241962 (PMC10534980; doi:10.3389/fendo.2023.1241962)
Supplement: Supplementary file 2 [file Table_1.docx]

Supplementary Material

Article Title

Wen-xin Ma^1^, Xiao-wen Zhang^1^, Ruo-tong Zhao^2^, Yang Tang^2^, Xiao-yun Zhu^3^, Long-kun Liu^2^, Ming-yuan Xu^2^, Ge Wang^4^, Pei-yue Peng^2^, Jian-ping Liu^1,5^, and Zhao-lan Liu^1*^

^1^Centre for Evidence-Based Chinese Medicine, Beijing University of Chinese Medicine, Beijing, China

^2^Beijing University of Chinese Medicine, Beijing, China

^3^Guang’anmen Hospital, China Academy of Chinese Medical Sciences, Beijing, China

^4^Guang’anmen Hospital South Campus, China Academy of Chinese Medical Sciences, Beijing, China

^5^The National Research Center in Complementary and Alternative Medicine (NAFKAM), Department of Community Medicine, Faculty of Health Science, UiT The Arctic University of Tromsø, Tromsø, Norway

*** Correspondence:** Zhao-lan Liu: lzl1019@163.com

# Supplementary Data

## Table 1 Search strategies

| **Databases/Registries** | **Search strategies** |
| --- | --- |
| **Pubmed** | #1 Hyperthyroidism [MeSH Terms] OR Graves Disease [MeSH Terms] OR Hyperthyroidism [Title/Abstract] OR Graves disease [Title/Abstract] OR Toxic diffuse goit* [Title/Abstract] OR diffuse goit* [Title/Abstract] OR hyperthyreosis [Title/Abstract] OR thyroid hyperfunction [Title/Abstract] OR thyrotoxicosis [Title/Abstract] OR toxic goit* [Title/Abstract] OR Hyperthyroid* [Title/Abstract]  #2 danzhixiaoyaosan[All Fields] OR dan-zhi-xiao-yao-san[All Fields] OR danzhixiaoyao[All Fields] OR 'dan zhi xiao yao'[All Fields] OR 'danzhi xiaoyao'[All Fields] OR jiaweixiaoyaosan[All Fields] OR 'jia-wei-xiao-yao-san'[All Fields] OR jiaweixiaoyaowan[All Fields] OR 'jia wei xiao yao'[All Fields] OR 'jiawei xiaoyao'[All Fields] OR dzxys[All Fields] OR jwxys[All Fields]  #3 #1 AND #2 |
| **Cochrane Library** | #1 MeSH descriptor: [Hyperthyroidism] explode all trees  #2 MeSH descriptor: [Graves Disease] explode all trees  #3 danzhixiaoyaosan  #4 Dan-Zhi-Xiao-Yao-San  #5 danzhixiaoyaowan  #6 dan zhi xiao yao  #7 danzhi xiaoyao  #8 jiaweixiaoyaosan  #9 Jia-Wei-Xiao-Yao-San  #10 jiaweixiaoyaowan  #11 jia wei xiao yao  #12 jiawei xiaoyao  #13 DZXYS  #14 JWXYS  #15 #3 OR #4 OR #5 OR #6 OR #7 OR #8 OR #9 OR #10 OR #11 OR #12 OR #13 OR #14  #16 #1 OR #2  #17 #15 AND #16 |
| **Embase** | #1 'hyperthyroidism'/exp OR 'feline hyperthyroidism' OR 'hyperthyreoidism' OR 'hyperthyreosis' OR 'hyperthyroid function' OR 'hyperthyroidea' OR 'hyperthyroidism' OR 'hyperthyroidosis' OR 'thyroid gland hyperfunction' OR 'thyroid hyperfunction' OR 'thyroideal hyperfunction' OR 'graves disease'/exp OR 'graves disease' OR 'graves hyperthyroidism' OR 'graves` disease' OR 'graves`s disease' OR 'basedow disease' OR 'basedow syndrome' OR 'disease, graves basedow' OR 'exophthalmic goiter' OR 'exophthalmic goitre' OR 'goiter, exophthalmic' OR 'graves basedow disease' OR 'graves syndrome' OR 'hyperthyroidism, exophthalmic' OR 'toxic goiter'/exp OR 'diffuse toxic goiter' OR 'goiter, toxic' OR 'struma, toxic' OR 'toxic goiter' OR 'toxic goitre' OR 'toxic multinodular goiter' OR 'toxic nodular goiter' OR 'toxic struma'  #2 danzhixiaoyaosan OR 'dan zhi xiao yao san' OR danzhixiaoyaowan OR 'dan zhi xiao yao' OR 'danzhi xiaoyao' OR jiaweixiaoyaosan OR 'jia wei xiao yao san' OR jiaweixiaoyaowan OR 'jia wei xiao yao' OR 'jiawei xiaoyao' OR dzxys OR jwxys  #3 #1 AND #2 |
| **Web of Science（Medline）** | #1 TS=('hyperthyroidism'/exp OR 'hyperthyroidism' OR 'hyperthyreosis' OR 'hyperthyroid function' OR 'hyperthyroidia' OR 'hyperthyroidism' OR 'hyperthyroidisis' OR 'thyroid gland hyperfunction' OR 'thyroid hyperfunction' OR 'thyroideae hyperfunction' OR 'graves disease'/exp OR 'graves disease' OR 'graves hyperthyroidism' OR 'graves` disease' OR 'graves`s disease' OR 'basedow disease' OR 'basedow syndrome' OR 'disease, graves basedow' OR 'exophthalmic goiter' OR 'exophthalmic goitre' OR 'goiter, exophthalmic' OR 'graves basedow disease' OR 'graves syndrome' OR 'hyperthyroidism, exophthalmic')  #2 TI=('danzhixiaoyaosan' OR 'Dan-Zhi-Xiao-Yao-San' OR 'danzhixiaoyaowan' OR 'dan zhi xiao yao' OR 'danzhi xiaoyao' OR 'jiaweixiaoyaosan' OR 'Jia-Wei-Xiao-Yao-San' OR 'jiaweixiaoyaowan' OR 'jia wei xiao yao' OR 'jiawei xiaoyao' OR 'DZXYS' OR 'JWXYS') OR AB=('danzhixiaoyaosan' OR 'Dan-Zhi-Xiao-Yao-San' OR 'danzhixiaoyaowan' OR 'dan zhi xiao yao' OR 'danzhi xiaoyao' OR 'jiaweixiaoyaosan' OR 'Jia-Wei-Xiao-Yao-San' OR 'jiaweixiaoyaowan' OR 'jia wei xiao yao' OR 'jiawei xiaoyao' OR 'DZXYS' OR 'JWXYS')  #3 #1 AND #2 |
| **China National Knowledge Infrastructure**  **（CNKI）** | #1 SU=('甲状腺功能亢进' + '甲状腺机能亢进' + '甲亢' + 'Graves病' + 'Graves disease' + '格雷夫斯' + '毒性弥漫性甲状腺肿' + '弥漫性毒性甲状腺肿' )  #2 SU=('甲状腺功能亢进' + '甲状腺机能亢进' + '甲亢' + 'Graves病' + 'Graves disease' + '格雷夫斯' + '毒性弥漫性甲状腺肿' + '弥漫性毒性甲状腺肿' )  #3 #1 OR #2 |
| **Wanfang Database** | #1 主题: ("甲状腺功能亢进" or "甲状腺机能亢进" or "甲亢" or "Graves" or "Graves'" or "格雷夫斯" or "毒性弥漫性甲状腺肿" or "弥漫性毒性甲状腺肿")  #2 主题: ("丹栀逍遥" or "加味逍遥" or "八味逍遥")  #3 #1 OR #2 |
| **CQVIP** | #1 M = (“甲状腺功能亢进” or “甲状腺机能亢进” or “甲亢” or “Graves” or “Graves’ disease” or “格雷夫斯” or “毒性弥漫性甲状腺肿” or “弥漫性毒性甲状腺肿”)  #2 R = (“甲状腺功能亢进” or “甲状腺机能亢进” or “甲亢” or “Graves病” or “Graves’ disease” or “格雷夫斯” or “毒性弥漫性甲状腺肿” or “弥漫性毒性甲状腺肿”)  #3 #1 OR #2  #4 M = (“丹栀逍遥” or “加味逍遥” or “八味逍遥”)  #5 R = (“丹栀逍遥” or “加味逍遥” or “八味逍遥”)  #6 #4 OR #5  #7 #3 AND #6 |
| **SinoMed** | #1: "甲状腺功能亢进"[常用字段:智能] OR "甲状腺机能亢进"[常用字段:智能] OR "甲亢"[常用字段:智能] OR "Graves"[常用字段:智能] OR "Graves’ disease "[常用字段:智能] OR "格雷夫斯"[常用字段:智能] OR "毒性弥漫性甲状腺肿"[常用字段:智能] OR "弥漫性毒性甲状腺肿"[常用字段:智能]  #2: "丹栀逍遥"[常用字段:智能] OR "加味逍遥"[常用字段:智能] OR "八味逍遥"[常用字段:智能]  #3: (#1) AND (#2) |
| **The World Health Organization International Clinical Trials Registry Platform (ICTRP)** | #1 Title: Hyperthyroidism OR Graves Disease  #2 Intervention: danzhixiaoyaosan OR dan-zhi-xiao-yao-san OR danzhixiaoyao OR 'dan zhi xiao yao' OR 'danzhi xiaoyao' OR jiaweixiaoyaosan OR 'jia-wei-xiao-yao-san' OR jiaweixiaoyaowan OR 'jia wei xiao yao' OR 'jiawei xiaoyao' OR dzxys OR jwxys  #3: (#1) AND (#2) |
| **ClinicalTrials.gov** | #1 Condition or disease: Hyperthyroidism OR Graves Disease  #2 Intervention/treatment: danzhixiaoyaosan OR dan-zhi-xiao-yao-san OR danzhixiaoyao OR 'dan zhi xiao yao' OR 'danzhi xiaoyao' OR jiaweixiaoyaosan OR 'jia-wei-xiao-yao-san' OR jiaweixiaoyaowan OR 'jia wei xiao yao' OR 'jiawei xiaoyao' OR dzxys OR jwxys  #3 #1 AND #2 |
| **The Chinese Clinical Trial Registry** | #1 研究疾病名称: 甲状腺 OR Graves病  #2 干预措施：逍遥  #3 #1 AND #2 |

## Table 2 Herbal components and dosage

| **Type of study** | **Author (Year）** | **Herbal components and dosage** | **Administration** |
| --- | --- | --- | --- |
| Randomized controlled trial | Wang SL^[1]^  (2011) | *Cortex Moutan* (Mudanpi) 15g, *Gardeniae Fructus* (Zhizi) 10g, *Radix Bupleuri* (Chaihu) 10g, *Angelicae Sinensis Radix* (Danggui) 10g, *Poria Cocos(Schw.) Wolf*. (Fuling) 15g, *Menthae Herba* (Bohe) 10g, *Atractylodes Macrocephala Koidz.* (Baizhu) 10g, *Paeoniae Radix Alba* (Baishao) 15g, *Ostrea gigas tnunb* (Shengmuli) 25g, *Prunellae Spica* (Xiakucao) 20g, *Litchi Semen* (Lizhihe) 15g, *Figwort Root* (Xuanshen) 15g, *Citri Reticulatae Semen* (Juhe) 15g, *Fritillariae Thunbrgii Bulbus* (Zhebeimu) 15g, *licorice (Shenggancao)* 9g, | Twice a day |
|  | Tang YL^[2]^  (2012) | *Radix Bupleuri* (Chaihu) 15g, *Codonopsis Radix* (Dangshen) 30g, *Cortex Moutan* (Mudanpi) 12g, *Gardeniae Fructus* (Zhizi) 18g, *Prunellae Spica* (Xiakucao) 12g, *Paeoniae Radix Alba* (Baishao) 15g, *Atractylodes Macrocephala Koidz*. (Baizhu) 15g, *Poria Cocos(Schw.) Wolf.* (Fuling) 15g, *Forsythiae Fructus* (Lianqiao) 30g, *Belamcandae Rhizome* (Shegan) 12g, *licorice* (Zhigancao) 9g, | Twice a day |
|  | Liu SY et al. ^[3-5]^  (2012+2012+2016) | *Radix Bupleuri* (Chaihu) 15g, *Cortex Moutan* (Mudanpi) 15g, *Gardeniae Fructus* (Zhizi) 15g, *Scutellariae Radix* (Huangqin) 15g, *Lycii Cortex* (Digupi) 15g, *Paeoniae Radix Alba* (Baishao) 15g, *Angelicae Sinensis Radix* (Danggui) 15g, *Atractylodes Macrocephala Koidz*. (Baizhu) 15g, *Menthae Herba* (Bohe) 15g, *Platycladi Semen* (Baiziren) 15g, *Chrysanthemi Flos* (Juhua) 15g, *Ophiopogon japonicus* (Maidong) 15g, *Ziziphi Spinosae Semen* (Suanzaoren) 20g, *Uncariae Ramulus Cumuncis* (Gouteng) 20g, *licorice* (Zhigancao) 6g | Three times a day |
|  | Huang FX et al.^[6]^  (2013) | *Radix Bupleuri* (Chaihu) 12g, *Paeoniae Radix Alba* (Baishao) 12g, *Angelicae Sinensis Radix* (Danggui) 12g, *Atractylodes Macrocephala Koidz*. (Baizhu) 15g, *Poria Cocos(Schw.) Wolf.* (Fuling) 15g, *Menthae Herba* (Bohe) 6g, *Zingiber Officinale Roscoe* (Shengjiang) 6g, *licorice* (Zhigancao) 6g, *Cortex Moutan* (Mudanpi) 10g, *Gardeniae Fructus* (Zhizi) 10g, *Radix Salviae* (Danshen) 20g, *Artemisiae Scopariae Herba* (Yinchen) 15g, *Prunellae Spica* (Xiakucao) 15g | Twice a day |
|  | Guo J^[7]^  (2015) | Cortex Moutan (Mudanpi) 10g, Gardeniae Fructus (Zhizi) 10g, *Radix Bupleuri* (Chaihu) 8g, *Angelicae Sinensis Radix* (Danggui) 10g, *Paeoniae Radix Alba* (Baishao) 10g, *Poria Cocos(Schw.) Wolf.* (Fuling) 15g, *Atractylodes Macrocephala Koidz.* (Baizhu) 10g | Twice a day |
|  | Qiu ZQ^[8]^  (2015) | *Radix Bupleuri* (Chaihu) 30g, *Angelicae Sinensis Radix* (Danggui) 30g, *Poria Cocos(Schw.) Wolf.* (Fuling) 30g, *Atractylodes Macrocephala Koidz.* (Baizhu) 30g, *Cortex Moutan* (Mudanpi) 15g, *Gardeniae Fructus* (Zhizi) 15g, *licorice* (Gancao) 6g | Twice a day |
|  | Li MY^[9]^  (2016) | *Radix Bupleuri* (Chaihu) 15g, *Cortex Moutan* (Mudanpi) 15g, *Gardeniae Fructus* (Zhizi) 15g, *Scutellariae Radix* (Huangqin) 15g, *Lycii Cortex* (Digupi) 15g, *Paeoniae Radix Alba* (Baishao) 15g, *Angelicae Sinensis Radix* (Danggui) 15g, *Atractylodes Macrocephala Koidz.* (Baizhu) 15g, *Menthae Herba* (Bohe) 15g, *Platycladi Semen* (Baiziren) 15g, *Chrysanthemi Flos* (Juhua) 15g, *Ophiopogon japonicus* (Maidong) 15g, *Ziziphi Spinosae Semen* (Suanzaoren) 20g, *Uncariae Ramulus Cumuncis* (Gouteng) 20g, *licorice* (Zhigancao) 6g | Three times a day |
|  | Wu MY^[10]^  (2017) | *Cortex Moutan* (Mudanpi) 15g, *Gardeniae Fructus* (Zhizi) 10g, *Radix Bupleuri* (Chaihu) 15g, *Angelicae Sinensis Radix* (Danggui) 10g, *Paeoniae Radix Alba* (Baishao) 10g, *Poria Cocos(Schw.) Wolf.* (Fuling) 15g, *Atractylodes Macrocephala Koidz.* (Baizhu) 10g, *Scutellariae Radix* (Huangqin) 10g, *licorice* (Gancao) 10g, *Prunellae Spica* (Xiakucao) 15g, *Forsythiae Fructus* (Lianqiao) 10g, *Ranunculus ternatus Thunb* (Maozhuacao) 30g, *Rehmannia glutinosa Libosch* (Shengdi) 15g, *Fritillariae Thunbrgii Bulbus* (Zhebeimu) 15g, *Citri Reticulatae Semen* (Juhe) 15g | Twice a day |
|  | Zhang LL^[11]^  (2017) | *Radix Bupleuri* (Chaihu) 15g, *Cortex Moutan* (Mudanpi) 15g, *Gardeniae Fructus* (Zhizi) 15g, *Scutellariae Radix* (Huangqin) 15g, *Lycii Cortex* (Digupi) 15g, *Paeoniae Radix Alba* (Baishao) 15g, *Angelicae Sinensis Radix* (Danggui) 15g, *Atractylodes Macrocephala Koidz*. (Baizhu) 15g, *Menthae Herba* (Bohe) 15g, *Chrysanthemi Flos* (Juhua) 15g, *Ziziphi Spinosae Semen* (Suanzaoren) 20g, *Platycladi Semen* (Baiziren) 15g, *Ophiopogon japonicus* (Maidong) 15g, *Uncariae Ramulus Cumuncis* (Gouteng) 20g, *licorice* (Zhigancao) 6g | Twice a day |
|  | Li JN^[12]^  (2018) | *Cortex Moutan* (Mudanpi) 15g, *Gardeniae Fructus* (Zhizi) 10g, *Angelicae Sinensis Radix* (Danggui) 15g, *Paeoniae Radix Alba* (Baishao) 15g, *Radix Bupleuri* (Chaihu) 15g, *Poria Cocos(Schw.) Wolf.* (Fuling) 15g, *Atractylodes Macrocephala Koidz.* (Baizhu) 15g, *Menthae Herba* (Bohe) 10g, *Arum Ternatum Thunb.* (Banxia) 15g, *Scutellariae Radix* (Huangqin) 15g, *Codonopsis Radix* (Dangshen) 15g, *Jujubae Fructus* (Dazao) 15g, *licorice* (Gancao) 10g, *Zingiber Officinale Roscoe* (Shengjiang) 15g | Three times a day |
|  | Tian LY et al.^[13]^  (2020) | *Cortex Moutan* (Mudanpi) 15g, *Gardeniae Fructus* (Zhizi) 12g, *Radix Bupleuri* (Chaihu) 15g, *Paeoniae Radix Alba* (Baishao) 12g, *Angelicae Sinensis Radix* (Danggui) 12g, *Poria Cocos(Schw.) Wolf.* (Fuling) 15g, *Atractylodes Macrocephala Koidz.* (Baizhu) 12g, *Aurantii Fructus Immaturus* (Zhishi) 12g, *licorice* (Zhigancao) 15g, *Scutellariae Radix* (Huangqin) 15g, *Coptidis Rhizoma* (Huanglian) 12g, *Prunellae Spica* (Xiakucao) 15g, *Fructus Ligustri Lucidi* (Nvzhenzi) 12g, *Ecliptae Herba* (Mohanlian) 15g | Twice a day |
|  | Shi LJ et al.^[14]^  (2020) | *Cortex Moutan* (Mudanpi) 20g, *Gardeniae Fructus* (Zhizi) 20g, *Paeoniae Radix Alba* (Baishao) 15g, *Menthae Herba* (Bohe) 15g, *Angelicae Sinensis Radix* (Danggui) 10g, *Radix Bupleuri* (Chaihu) 10g, *Atractylodes Macrocephala Koidz.* (Baizhu) 10g, *Poria Cocos(Schw.) Wolf.* (Fuling) 10g, *Zingiberis Rhizoma* (Ganjiang) 6g, *licorice* (Gancao) 6g | Twice a day |
|  | Gao R et al.^[15]^  (2021) | *Cortex Moutan* (Mudanpi) 10g, *Gardeniae Fructus* (Zhizi) 10g, *Angelicae Sinensis Radix* (Danggui) 10g, *Paeoniae Radix Alba* (Baishao) 30g, *Radix Bupleuri* (Chaihu) 15g, *Poria Cocos(Schw.) Wolf.* (Fuling) 15g, *Atractylodes Macrocephala Koidz.* (Baizhu) 10g, *Prunellae Spica* (Xiakucao) 10g, *Cyperi Rhizoma* (Xiangfu) 10g, *Curcumae Radix* (Yujin) 10g, *Ziziphi Spinosae Semen* (Suanzaoren) 10g, *licorice* (Gancao) 5g, *Zingiber Officinale Roscoe* (Shengjiang) 5g | Twice a day |
| Case report | Chang CC^[16]^ (2010) | JWXYS (*Sun Ten Pharmaceutical Co. Ltd., Taiwan*) 2g in addition of *Fritillaria thunbergii* (Beimu) 0.5g, *Prunella vulgaris* (Xiakucao) 0.5g, *Ostrea gigas Thunberg* (Muli) 0.5g. JWXYS includes *Bupleurum chinense* (Chai Hu, *Radix Bupleurum chinensis*), *Atractylodes macrocephala* (Bai Zhu, *Rhizoma Atractylodis macrocephalae*), *Paeonia lactiflora* (Bai Shao, *Radix Paeoniae alba*), *Angelica sinensis* (Dang Gui, *Radix Angelica sinensis*), *Wolfiporia cocos* (Fu Ling, *Poria cocos*), *Glycyrrhiza uralensis* (Gan Cao, *Radix Glycyrrhizae*), *Mentha canadensis* (Bo He, *Herba Menthae*), *Paeonia suffruticosa* (Mu Dan Pi, *Cortex Moutan*), *Gardenia jasminoides* (Zhi Zi, *Fructus Gardeniae*) and *Zingiber officinale* (Sheng Jiang, *Rhizoma Zingiberis recens*). The relative ratios of the 10 components are Chai Hu 4: Bai Zhu 4: Bai Shao 4: Dang Gui 4: Fu Ling 4: Gan Cao 2: Bo He 2: Mu Dan Pi 2.5: Zhi Zi 2.5: Sheng Jiang 4. | Three times a day |
|  | Lin CH^[17]^ (2021) | JWXYS (*Sun Ten Pharmaceutical Co. Ltd.*, Taiwan) 2g in addition of *Fritillaria thunbergii* (Beimu) 0.5 g, *Prunella vulgaris* (Xiakucao) 0.5 g, *Ostrea gigas Thunberg* (Muli) 0.5g. | Three times a day |
|  | Xia XG^[18]^ (2009) | First visit: *Cortex Moutan* 12g, *Gardeniae Fructus* 15g, *Radix Bupleuri* 12g, *Angelicae Sinensis Radix* 15g, *Paeoniae Radix Alba* 15g, Atractylodes Macrocephala Koidz. 12g, *Poria Cocos(Schw.) Wolf.*15g, *Menthae Herba* 12g (decocted later), *Ziziphi Spinosae Semen* 12g, *Tribulus terrestris L* 15g, *Haliotis discus hannai* 30g (decocted first), *licorice* 6g. Sencond visit after 15 days: in addition of *Hedysarum Multijugum Maxim.* 30g, *Ophiopogon japonicus* 15g. Third visit after 40 days: Jiawei Xiaoyao Pills, 6g. | Three times a day |
| Pharmacological study | Wu XY^[19]^ (2021) | JWXYS in addition of *Anemone flaccida Fr. Schmidt* (Diwu). Original drug concentration 3.0 g/mL | 1.21g/kg, 3.84g/kg, 12.14g/kg per day |
|  | Bao CY et al.^[20]^ (2019) | *Cortex Moutan* 15g, *Gardeniae Fructus* 12g, *Radix Bupleuri* 12g, *Atractylodes Lancea (Thunb.)Dc.*12g, *Lycii Cortex* 12g, *Paeoniae Radix Alba* 10g, *Angelicae Sinensis Radix* 10g, *Atractylodes Macrocephala Koidz.* 10g, *Zingiber Officinale Roscoe* 5g, *licorice* 6g, *Platycladi Semen* 15 g, *Menthae Herba* 8g, *Ophiopogon japonicus* 15g, *Uncariae Ramulus Cumuncis* 12g, *Ziziphi Spinosae Semen* 16g | 11g/kg per day |
|  | Tan HZ et al.^[21-25]^ (2017) | *Radix Bupleuri* 15g, *Cortex Moutan* 15g, *Gardeniae Fructus* 15g, *Scutellariae Radix* 15g, *Lycii Cortex* 15g, *Paeoniae Radix Alba* 15g, *Angelicae Sinensis Radix* 15g, *Atractylodes Macrocephala Koidz.* 15g, *Menthae Herba* 15g, *Chrysanthemi Flos* 15g, *Ziziphi Spinosae Semen* 20g, Platycladi Semen 15g, *Ophiopogon japonicus* 15g, *Uncariae Ramulus Cumuncis* 20g, *licorice* 6g | 13.65g/kg, 27.3g/kg, 54.6g/kg per day |

# References

[1] Wang S L. Clinical research of Danzhi Xiaoyao Powder modified thismazole on hyperthyroidism. Master dissertation[D]. Guangzhou, China: Jinan University, 2011.

[2] Tang Y L. Clinical Observation of the Effect of Dan Zhi Xiao Yao Decoction on the Patients With Liver stagnation and spleen deficiency and heat of hashimoto's thyroiditis and Hyperthyroidism. Master dissertation[D]. Jinan, China: Shandong University of Traditional Chinese Medicine, 2012.

[3] Liu S Y. Clinical Observation on the treatment of blood glucose, blood lipid and depression in patients with hyperthyroidism (type of pathogenic fire derived from stagnation of liver-QI) by soothing the liver and puring fire. Master dissertation[D]. Chengdu, China: Chengdu University of Traditional Chinese Medicine, 2012.

[4] Zhang J J. Clinical observation on the method of soothing liver and clearing heat in the treatment of patients with hyperthyroidism (type of liver depression inducing fire). Master dissertation[D]. Chengdu, China: Chengdu University of Traditional Chinese Medicine, 2012.

[5] Fu X X, Li H Y, Kang X Y, et al. Clinical study on Danzhi compound in treating hyperthyroidism (type of liver depression inducing fire)[J]. Journal of Sichuan of Traditional Chinese Medicine, 2016, 34(03): 70-73.

[6] Huang F X, Chen C G, Wu M F. Clinical study of Danzhi Xiaoyao pill in treating hyperthyroidism induced liver injury [Article in Chinese][J]. Hebei Traditional Chinese Medicine, 2013, 35(02): 187-189.

[7] Guo J. Clinical Observation of the treatment of Graves' Disease by Using Dan Zhi xiaoyao powder. Master dissertation.[D]. Hubei University of Traditional Chinese Medicine, 2015.

[8] Qiu Z Q, Qian H Q. Clinical observation of Xiaoyao powder combined with western medicine in treating hyperthyroid cardiopathy [Article in Chinese][J]. Journal of Emergency in Traditional Chinese Medicine, 2015, 24(05): 916-917.

[9] Li M Y. Effects of Danzhi compound on the thyroid function in mice with Graves’ hyperthyroidism and patients with hyperthyroidism characterized with pathogentic fire derived from stagnation of liver-Qi. Master dissertation[D]. Chengdu university of traditional chinese medicine, 2016.

[10] Wu M Y. Clinical observation on effect of Danzhixiaoyao powder in the treatment of hyperthyroidism. Master dissertation[D]. Guangzhou, China: Guangzhou University of Traditional Chinese Medicine, 2017.

[11] Zhang L L. Liver Hydrophobic Heat Method Integrated Clinical Intervention for Patients with Hyperthyroidism. Master dissertation[D]. Chengdu, China: Chengdu University of Traditional Chinese Medicine, 2017.

[12] Li J N. Clinical study on the treatment of hyperthyroidism (liver-fire exuberance syndrome) by inhibiting yang and strengthening yin. Master dissertation[D]. Chengdu, China: Chengdu University of Traditional Chinese Medicine, 2018.

[13] Tian L Y, Zhang G L, He H, et al. Clinical trial of Danzhixiaoyao powder combined with Thiamazole in treating hyperthyroidism [Article in Chinese][J]. Hunan Journal of Traditional Chinese Medicine, 2020, 36(11): 62-64.

[14] Shi L J, Liu J, Zhang X R, et al. Clinical study of Danzhi Xiaoyao powder combined with methimazole in treating hyperthyroidism [Article in Chinese][J]. Shanxi Journal of Traditional Chinese Medicine, 2020, 41(09): 1276-1278+1300.

[15] Gao R, Hu J Z. Clinical Observation of Danzhi Xiaoyao Powder in the Treatment of Irate Exuberant Syndrome of Graves Disease. [Article in Chinese] [J]. Clinical Journal of Traditional Chinese Medicine, 2021, 33(04): 730-733.

[16] Chang C C, Huang S T. Is Traditional Chinese medicine effective for reducing hyperthyroidism?[J]. J Altern Complement Med, 2010, 16(11): 1217-20.

[17] Lin C H, Lin C P, Huang S T. Successful intervention with Chinese herbal medicine for hyperthyroidism: Two case reports and a literature review[J]. Explore (NY), 2021, 17(4): 344-350.

[18] Xia X G. Using Danzhi Xiaoyao powder to treat hyperthyroidism[Article in Chinese][J]. Sichuan Traditional Chinese Medicine, 2009, 27(06): 95.

[19] Wu X Y. Preliminary study on the efficacy and pharmacology of Jiawei Xiaoyao Pills in treating Graves' disease. Master dissertation[D]. Peking Union Medical College, 2021.

[20] Bao C Y, Zhou L Y, Yu X X, et al. Effects of propyl thiouracil combined with Danzhi compound on thyroid function in the progeny of mice with hyperthyroidism and pregnancy[J]. Journal of Guangzhou University of Chinese Medicine, 2019, 36(07): 1059-1063.

[21] Tan H Z. Study on dose-effect and time-effect relationship and mechanism of Danzhi Compound in treating Graves disease mice in the view of "liver". Master dissertation[D]. Chengdu university of traditional chinese medicine, 2017.

[22] Liu J. Effect of Danzhi Compound on oxidative stress response of liver injury in hyperthyroidism model mice. Master dissertation[D]. Chengdu university of traditional chinese medicine, 2017.

[23] Liu Q. Effects of Danzhi Compound on thyroid function, liver injury and oxidative stress in GD mice. Master dissertation[D]. Chengdu university of traditional chinese medicine, 2017.

[24] Huang Y W. Effect of Danzhi Compound on the expression of thyroid Caspase-3,Bcl-2 and Bax protein in mice with Graves' disease. Master dissertation[D]. Chengdu university of traditional chinese medicine, 2017.

[25] Jia Y L. Effects of Danzhi Compound on thyroid function and DNA oxidative damage of hepatocytes in hyperthyroidism model mice. Master dissertation[D]. Chengdu university of traditional chinese medicine, 2017.
